# Isothiocyanates (ITCs) 1-(Isothiocyanatomethyl)-4-phenylbenzene and 1-Isothiocyanato-3,5-bis(trifluoromethyl)benzene—Aldehyde Dehydrogenase (ALDH) Inhibitors, Decreases Cisplatin Tolerance and Migratory Ability of NSCLC

**DOI:** 10.3390/ijms23158644

**Published:** 2022-08-03

**Authors:** Jolanta Kryczka, Jakub Kryczka, Łukasz Janczewski, Anna Gajda, Andrzej Frączyk, Joanna Boncela, Beata Kolesińska, Ewa Brzeziańska-Lasota

**Affiliations:** 1 Department of Biomedicine and Genetics, Medical University of Lodz, 92-213 Lodz, Poland; ewa.brzezianska@umed.lodz.pl; 2 Institute of Medical Biology, Polish Academy of Sciences, 93-232 Lodz, Poland; jkryczka@cbm.pan.pl (J.K.); jboncela@cbm.pan.pl (J.B.); 3 Institute of Organic Chemistry, Faculty of Chemistry, Lodz University of Technology, 90-924 Lodz, Poland; lukasz.janczewski@p.lodz.pl (Ł.J.); anna.gajda@p.lodz.pl (A.G.); beata.kolesinska@p.lodz.pl (B.K.); 4 Institute of Applied Computer Science, Lodz University of Technology, 90-537 Lodz, Poland; andrzej.fraczyk@p.lodz.pl

**Keywords:** non-small-cell lung cancer, cisplatin resistance, aldehyde dehydrogenase, isothiocyanates, disulfiram, epithelial to mesenchymal transition

## Abstract

One of the main treatment modalities for non-small-cell lung cancer (NSCLC) is cisplatin-based chemotherapy. However, the acquisition of cisplatin resistance remains a major problem. Existing chemotherapy regimens are often ineffective against cancer cells expressing aldehyde dehydrogenase (ALDH). As such, there is an urgent need for therapies targeting ALDH-positive cancer cells. The present study compares the anticancer properties of 36 structurally diverse isothiocyanates (ITCs) against NSCLC cells with the ALDH inhibitor disulfiram (DSF). Their potential affinity to ALDH isoforms and ABC proteins was assessed using AutoDockTools, allowing for selection of three compounds presenting the strongest affinity to all tested proteins. The selected ITCs had no impact on NSCLC cell viability (at tested concentrations), but significantly decreased the cisplatin tolerance of cisplatin-resistant variant of A549 (A549CisR) and advanced (stage 4) NSCLC cell line H1581. Furthermore, long-term supplementation with ITC 1-(isothiocyanatomethyl)-4-phenylbenzene reverses the EMT phenotype and migratory potential of A549CisR to the level presented by parental A549 cells, increasing E-Cadherin expression, followed by decreased expression of ABCC1 and ALDH3A1. Our data indicates that the ALDH inhibitors DSF and ITCs are potential adjuvants of cisplatin chemotherapy.

## 1. Introduction

Since its introduction into clinical trials in 1971 and subsequent Food and Drug Administration approval in 1978, cisplatin represents a major landmark in the history of successful anti-cancer therapeutics. It has changed the management of several solid malignancies, including lung cancer, which remains the second-most-common cancer globally and the leading cause of cancer death [1]. Approximately 2.2 million of new cases of lung cancer are estimated to occur each year worldwide, with a mean 5-year survival rate of 22% [2,3].

One of the factors that contributes to such a low survival rate is the development of cisplatin resistance [4,5]. Cisplatin resistance is very complicated because of the multifactorial composition of several mechanisms including DNA repair, induction of anti-apoptotic signals, and the active efflux of drugs from the cell cytoplasm. Additionally, cisplatin-resistant cells were proven to undergo Epithelial to Mesenchymal Transition (EMT), which plays a substantial role in cancer progression and metastasis, increasing cancer cell motility and invasiveness [5]. EMT renders cancer cells virtually impervious to the majority of anticancer drugs, decreasing cell proliferation and directly influencing the expression of ABC-family transporters, which are involved in multidrug resistance [6,7].

Recently, aldehyde dehydrogenase (ALDH) was confirmed to play a role in drug resistance in lung cancer [8]. A bioinformatic analysis of metabolic enzymes found cisplatin-resistant NSCLC to express ALDH [9]. ALDH family members are cytosolic or mitochondrial isoenzymes that are responsible for oxidizing intracellular aldehydes. They play a role in the oxidation of retinol to retinoic acid in early stem cell differentiation [10]. Several of the 19 genes known to encode the ALDH family, such as ALDH1A1, ALDH1A2, ALDH1A3, ALDH1A7, ALDH3A1, ALDH4A1, ALDH5A1, ALDH6A1, and ALDH9A1, are considered the cancer stem cells (CSC) markers involved in drug resistance [11,12,13]. Notably, currently used chemotherapy regimens were shown to be ineffective against ALDH-positive, cisplatin-resistant cancer cells [8]. Importantly, while ALDH1 activity has been reported in a number of NSCLC cell lines and tumor samples, its role in chemotherapy resistance remains unclear [4]. Therefore, it may be beneficial to investigate therapies targeting drug-resistant cancer cells expressing ALDH [8]. One group of small-molecule ALDH inhibitors that may be promising candidates for anti-cancer therapy is the isothiocyanates (ITCs).

Isothiocyanates are commonly known for their anti-cancer properties. They are low-molecular-weight, natural, organic composites characterized by a pungent odor [14], with the general formula R–NCS. They are found in cruciferous vegetables, such as radish, horseradish, wasabi, broccoli, or Brussels sprouts [15,16] and are produced by the reaction of glucosinolates with myrosinases [17,18,19,20]. In addition to natural ITCs such as benzyl isothiocyanate [21,22], phenethyl isothiocyanate [23,24] or best-tested sulforaphane (SFN) [25,26,27,28,29,30], synthetic counterparts of ITCs have been produced by modification with a fluorine atom [31,32], phosphorous group [33,34], or other functional groups [35]. ITCs are used in organic chemistry as substrates to synthesize inter alia heterocyclic compounds or thioamides [36]. However, these compounds are best-known for their anticancer activity [27,37,38,39,40,41] and antibacterial activity [42,43,44].

In prostate cancer, ITCs decreased the concentration of the anti-apoptotic proteins Bcl-2 (B-cell lymphoma 2) and Bcl-xl (B-cell lymphoma-extra large) and increased the expression of pro-apoptotic proteins Bax (BCL2 Associated X, Apoptosis Regulator) and activate caspases [45]. Additionally, they decreased the activity of apoptosis inhibitors such as cIAP1 (cellular inhibitor of apoptosis protein-1), cIAP2 (cellular inhibitor of apoptosis 2), and XIAP (X-linked inhibitor of apoptosis protein) and induced Apaf1 (Apoptotic Peptidase Activating Factor 1) protein activity [46]. In addition, in human embryonic kidney cell line HEK293, ITCs were found to suppress transcription of histone deacetylases (HDACs), thus deregulating apoptosis- and differentiation-controlling mechanisms [47]. Furthermore, several studies have confirmed that ITCs suppress both the angiogenesis of human umbilical vein endothelial cells (HUVECs) [48] and metastasis of B16F-10 melanoma [33,49]. Additionally, benzyl isothiocyanates BITCs were proven to inhibit the phosphorylation activity of three major mitogen-activated protein kinases (MAPKs), ERK1/2, p38, and p-JNK1/2, thus presenting direct anti-metastatic activities in SK-Hep1 cells [50].

To improve the current therapeutic efficacy of this cisplatin, alternative strategies are needed to overcome resistance. In the present study, 36 structurally different ITCs (**1**–**36**) were synthesized and tested in vitro using lines of lung cancer cells and their cisplatin-resistant variants A549, A549CisR, and NCI-H1581. The protein level and function of two isoforms of ALDH were noted, these being potential markers of cisplatin resistance. Furthermore, the effects of targeting ALDH3A1 and ALDH7A1 by chemical inhibition were assessed in terms of their ability to re-sensitize resistant lung tumor cells to the cytotoxic effects of cisplatin. Moreover, as multiple ITCs were proven to present many, ostensibly not related, anticancer properties, due to different ITCs cellular targets, ITCs 1-(isothiocyanatomethyl)-4-phenylbenzene (named **19**) was screened in regards to possible antimetastatic abilities. ITCs 19 supplementation significantly decreases proteolytic abilities and in the aftermath the invasiveness of A549CisR, thus highlighting multifactorial anticancer properties.

## 2. Results

### 2.1. Characteristics of the Cisplatin-Resistant A549 Cell Line

**Figure 1:**
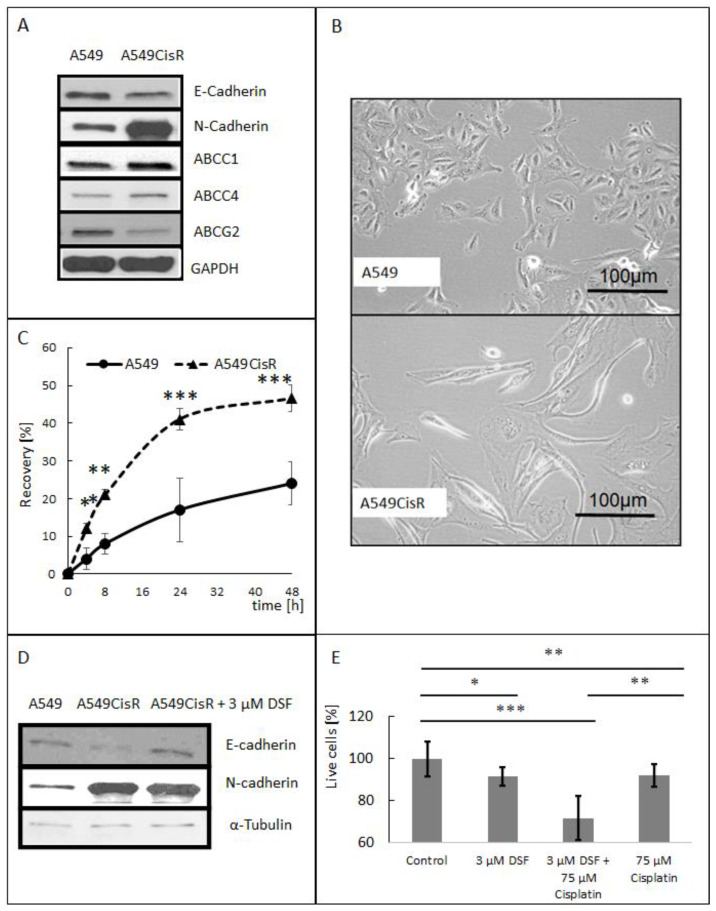
Characteristics of the cisplatin-resistant A549 cell line and DSF reversion of cisplatin resistance. (**A**) Western blot analysis of EMT markers and ABC proteins, performed in standard reducing SDS PAGE conditions. (**B**) Cell morphology (phase contrast microscopy). (**C**) Wound healing such as analysis of resistant-cell migration. A549 and A549CisR cells were seeded on a six-well plate and grown to confluence. Next, a wound was created and rinsed twice with PBS. New full medium was added. Wounded area was visualized after 0, 4, 8, 24, and 48 h using an OLYMPUS IX53 microscope and calculated by ImageJ software, *n* = 4, * *p* < 0.05; ** *p* < 0.005; *** *p* < 0.001, NS—not statistically significant. (**D**) DSF treatment reverses cisplatin resistance. Standard SDS-PAGE Western blot analysis of EMT markers. The A549CisR cells were treated with 3 µM DSF (48 h), and cell lysates were obtained using M-PER Mammalian Protein Extraction Reagent #78501 as described in Materials and Methods. (**E**) To determine the impact of DSF on cell viability, A549CisR cells were seeded on 96-well plates and treated with 75 µM cisplatin, 3 µM DSF, and 3 µM DSF 75 µM cisplatin. After 48 h, cell viability was tested using WST-1 assay (ScienCell, Research Lab., Carlsbad, CA, USA); *n* = 3, * *p* < 0.05; ** *p* < 0.005; *** *p* < 0.001, NS—not statistically significant.

A cisplatin-resistant variant of A549 (named A549CisR) was created by constant culturing in increasing cisplatin concentrations (1–10 µM), resulting in an IC_50_ value of 150 µM, compared to 75 µM for A549 (data not shown). A549CisR acquired a mesenchymal-like phenotype manifested by upregulation of mesenchymal marker N-cadherin, with simultaneous repression of epithelial marker E-cadherin (Figure 1A), as noted previously [51,52]. The degree of mesenchymal properties acquired via EMT varied between cells from an epithelial-like status, through a mixed epithelial/mesenchymal (E/M hybrid) form to a strongly mesenchymal phenotype. The hybrid and mesenchymal cells exhibited increased invasive features and circulating tumor cell (CTC) characteristics, suggesting that EMT plays an important role during metastatic dissemination [53]. The A549CisR EMT phenotype was followed by changes in ALDH3A1, ALDH7A1, and ABC protein expression (Figure 1A). A549CisR presented significantly higher ALDH3A1 (stem cell marker [10]), ALDH7A1, ABCC1, and ABCC4 expression and lower expression of ABCG2 compared to A549. Interestingly, ALDH1A1 expression did not differ (data not shown). Furthermore, we noticed changes in cell morphology, A549CisR cells became larger, spindle-shaped, and less densely packed than the parental A549 (Figure 1B). The cisplatin-induced EMT model mimics a natural shift toward higher aggression, and increased the migratory ability and metastasis obtained by chemo-resistant cancer cells during cancer progression [5,54].

Regarding the effect of cisplatin resistance related changes on NSCLC migration, A549CisR presented a higher 2D migration rate (observed in wound healing assay) than the parental sensitive variant (A549) (Figure 1C), even though this variant is considered to be a fairly aggressive NSCLC model [55]. This further demonstrates the increased metastatic potential associated with cisplatin resistance.

### 2.2. ALDH Inhibitor—Disulfiram Impact on Suppression of Cisplatin Resistance

The study also analyzed the impact of disulfiram (DSF), a well-known ALDH inhibitor, on A549 and A549CisR. DSF has been used to control alcohol abuse for many decades; however, it has recently been found to have strong anticancer activity both in vitro and in cancer xenografts [56]. DSF treatment (3 µM, 48 h) slightly reduces the mesenchymal phenotype of the cisplatin-resistant variant of A549, restoring the expression of E-cadherin, an epithelial marker (Figure 1D), to the level observed in parental A549. However, the phenotype was not fully restored, as the expression of N-cadherin, a mesenchymal marker, remained unchanged. Importantly, DSF partially re-sensitized A549CisR cells to cisplatin treatment (Figure 1E). Supplementation with 3 µM DSF followed by 75 µM cisplatin (24 h) significantly reduced the tolerance of A549CisR to cisplatin treatment. DSF impact on A549CisR viability is presented in Supplementary Materials (Figure S1).

### 2.3. Analysis of Cisplatin Resistance in NSCLC Cells Based on Changes in mRNA Expression of ALDH Family Proteins, EMT Marker, and ABC Proteins According to GEO Data

**Figure 2:**
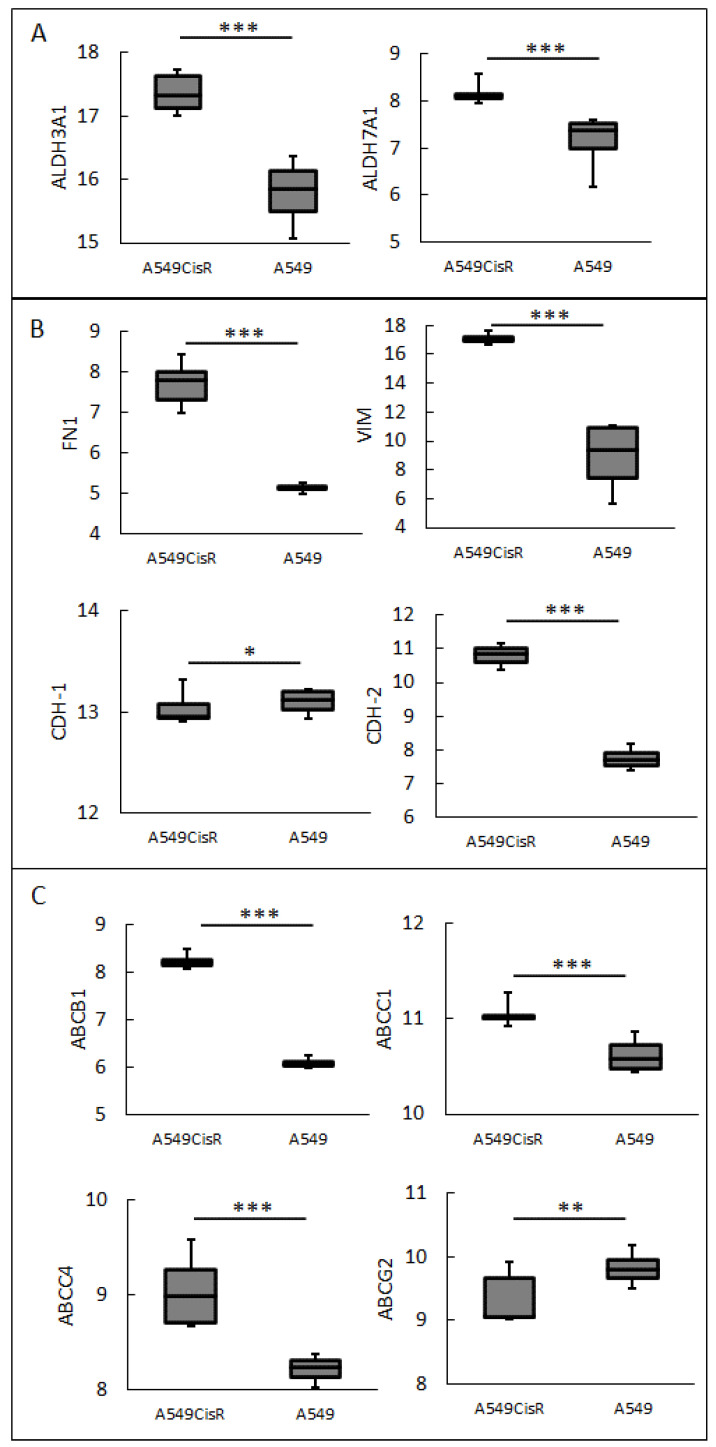
Cisplatin-resistance-related changes in mRNA expression of ALDH family proteins (**A**), EMT markers (**B**), and ABC proteins (**C**). NSCLC cell line A549 and its cisplatin-resistant variant A549CisR mRNA levels were analyzed using microarray dataset GSE108214, acquired from the public Gene Expression Omnibus (GEO) databases [59]. Normality test (Shapiro–Wilk) was performed, followed by the Mann–Whitney U test; * *p* < 0.05; ** *p* < 0.005; *** *p* < 0.001, not statistically significant—no indicator.

Delivery of cisplatin-resistant NSCLC cells is considered the standard approach (Materials and Methods section). However, to confirm whether the phenotypical changes occurring in the A549CisR variant are universal, the differences in mRNA expression between A549 and A549CisR were compared using the Gene Expression Omnibus GSE108214 database (Figure 2). A549CisR demonstrated significantly higher ALDH3A1 (stem cell marker) [10] and ALDH7A1 mRNA expression compared to parental A549, similarly to those obtained by our Western blot results. Interestingly, no statistically significant changes were observed in the mRNA expression of the most well-known stem cell marker, ALDH1A1 (Figure 2A) [10].

In addition, A549 demonstrated significant upregulation of all major EMT markers, including vimentin (VIM), fibronectin (FN1), and N-Cadherin (CDH-2) (Figure 2B), with no repression of epithelial E-Cadherin (CDH-1). This suggests that acquisition of cisplatin resistance is accompanied by the development of an advanced (not yet fully completed) hybrid EMT phenotype, with strong migratory potential. Recently, EMT was confirmed to be an important regulator of several ABC proteins, as the promoters of ABC transporters contain several binding sites for EMT-inducing transcription factors such as: Twist, Snail, and ZEB. Thus, this leads to increased ABC protein mRNA expression and increased broad, multidrug resistance [7,57,58]. Analysis of the GSE108214 data set confirmed that the mRNA expression of ABCB1, ABCC1, and ABCC4 by cisplatin-resistant variants of A549 (A549CisR) was significantly upregulated, whereas ABCG2 was downregulated (Figure 2C).

### 2.4. Assessment of Affinity of Isothiocyanates to Chosen ADHD and ABC Family Protein

In vivo, DSF is rapidly metabolized to diethyldithiocarbamate (DDTC), which is further converted to *S*-methyl-*N*,*N*-diethyldithiocarbamate (Me-DDTC) and *S*-methyl-*N*,*N*-diethylthiocarbamate (DETC). Subsequently, P450 catalyzes the oxidation of DETC, and Me-DDTC produces DETC-sulfoxide (DETC-SO) and *S*-methyl-*N*,*N*-diethyldithiocarbamate-sulfoxide (Me-DDTC-SO) and -sulfone (Me-DTC-SO_2_), metabolites that are most likely directly involved in ALDH inhibition. Importantly, when downstream steps of DSF metabolism are blocked by a chemical P450 inhibitor, liver ALDH remains uninhibited, confirming that it is the metabolites of DSF that are the true inhibitors of ALDH in vivo [60].

**Figure 3:**
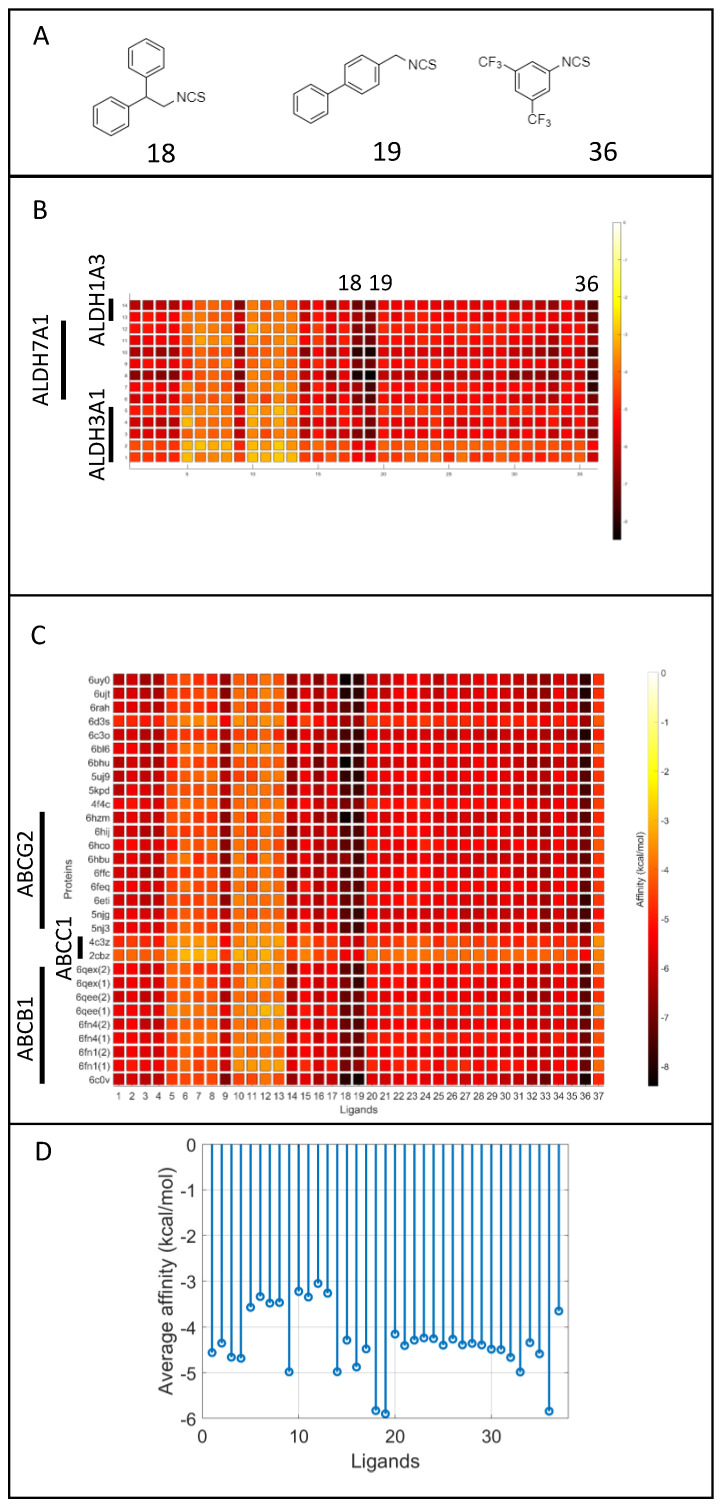
Molecular modeling of isothiocyanate (ICT) affinity to the tested ALDH and ABC proteins. (**A**) Chemical structures of three chosen ICTs: **18**, **19**, and **36**. (**B**) ICT affinity to the models of ALDH isoforms and (**C**) ABC proteins obtained from RCSB Protein Data Bank. (**D**) Average affinity of ICTs to all selected proteins, calculated as average of all affinity value to all structural models of each protein.

Therefore, the study examined the effects of isothiocyanate DSF analogues (ITCs) synthesized for the purpose of the study. In silico molecular modeling allowed the selection of the three most promising compounds: (2-isothiocyanatoethane-1,1-diyl)dibenzene named **18**, 1-(isothiocyanatomethyl)-4-phenylbenzene named **19**, and 1-isothiocyanato-3,5-bis(trifluoromethyl)benzene named **36** (Figure 3A); these presented the highest affinity to the several available models (RCSB Protein Data Bank) of ALDH isoforms, including ALDH3A1 and ALDH7A1, which were upregulated in the cisplatin-resistant variant of A549 (Figure 3B), and the ABC proteins involved in drug resistance (ABCB1, ABCC1, and ABCG2) (Figure 3C). Among the tested ITCs, the highest average affinity to all selected proteins was demonstrated by **18**, **19**, and **36** (Figure 3D).

### 2.5. Impact of ITCs on the Reversion of Cisplatin Resistance, EMT Phenotype and Cell Migration

**Figure 4:**
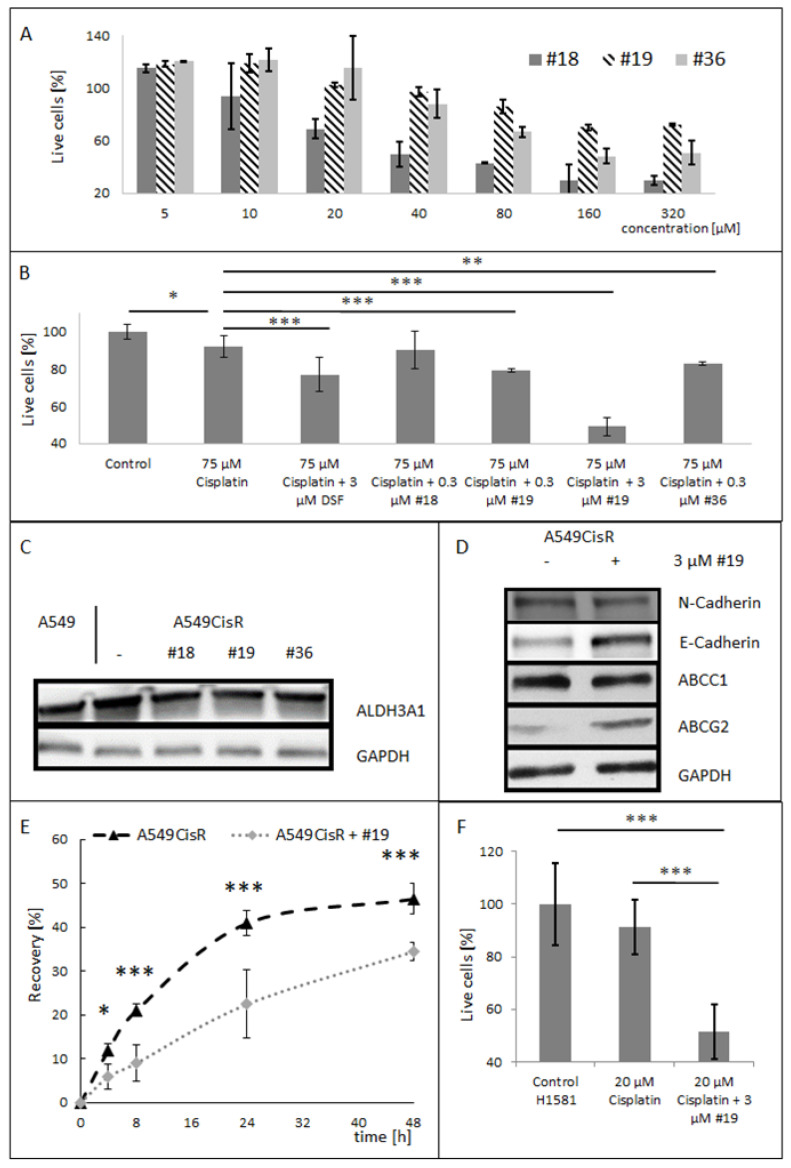
Induction of cisplatin-resistance reversion by isothiocyanates. (**A**) The impact of isothiocyanate concentration on A549 cell viability—WST-1 assay after 24 h supplementation with compounds **18**, **19**, or **36** (ScienCell, Research Lab., Carlsbad, CA, USA). (**B**) Reversion of cisplatin resistance (WST-1 assay). (**C**) Downregulation of ALDH3A1 by ITCs. Long-term (3 µM, 15 days) impact of compound **19** on EMT marker and ABC protein levels. (**D**) Standard SDS-PAGE Western blot. (**E**) Wound-healing-like analysis of the impact of compound **19** on cisplatin-resistant NSCLC cell migration. A549CisR and A549CisR supplemented with compound **19** (3 µM, 15 days) cells were seeded on a six-well plate, grown to confluence. A wound was made and rinsed twice with PBS. New full medium was added. The wounded area was visualized after 0, 4, 8, 24, and 48 h using an OLYMPUS IX53 microscope and calculated by ImageJ software, *n* = 3. (**F**) ITC **19** increases cisplatin sensitization of advanced NSCLC cells NCI-H1581. * *p* < 0.05; ** *p* < 0.005; *** *p* < 0.001, not statistically significant—no marker.

The chosen isothiocyanates are well-tolerated by the A549 cell line across a broad concentration range for 24 h: the IC_50_ values were approximately 80 μM for ITC **18** but are not present for ITC **19** and are 360 μM for ITC **36**. The ITCs demonstrated no cytotoxic effect against A549 or A549CisR at concentrations of 0.1–5 µM (Figure 4A). However, **19** and **36** significantly repressed the cisplatin resistance of A549CisR cells at 10-fold lower concentrations (0.3 µM), similar to DSF itself (3 µM) (Figure 4B). No statistically significant changes in cisplatin resistance were observed for combined 0.3 µM ITC **18** and 75 µM cisplatin (Figure 4B). Additionally, ITCs 19 impact on cisplatin resistance repression was tested in 3 µM concentration, presenting nearly 50% higher sensitivity compared to the untreated, resistant variant A549 (Figure 4B).

As compound **19** presented the strongest cisplatin-resistance reversion abilities and the highest tolerability in both tested cell lines, it was chosen for further study of its long-term impact on cisplatin-resistant NSCLC cells when applied at the same concentration as DSF itself (3 µM). Fifteen-day supplementation with 3 µM ITC **19** resulted in significant repression of ALDH3A1 expression (similarly to **18** and **36**) (Figure 4C) suggesting the reversion of a stem-cell-like phenotype. Furthermore, compound **19** was found to partially reverse EMT phenotype, presenting increased expression of the epithelial marker E-cadherin, with no significant changes in N-cadherin expression (Figure 4D) (similar to DSF). Phenotype reversion was also accompanied by the acquisition of an ABC protein-expression pattern, characteristic of the non-resistant A549 parental variant (Figure 4D), i.e., a decrease in ABCC1 and an increase in ABCG2 expression.

Furthermore, acquisition of cisplatin resistance increased the migratory abilities of A549CisR cells, rendering them highly metastatic. However, treatment with ITC **19** (15 days, 3 µM) significantly lowered 2D migration rate, as indicated by wound healing assay (Figure 4E).

### 2.6. Isothiocyanate #19 Increases Cisplatin Sensitization of Advanced NSCLC Cells

As ITC **19** significantly reduces cisplatin resistance in A549CisR, it was also tested against NCI-H1581, a stage 4 NSCLC cell line (CRL-5878) (Figure 4F). NSCLC predominantly consists of adenocarcinoma (AC) and squamous cell carcinoma (SCC). H1581 represents the smallest subfraction (10%) of NSCLC: a large cell carcinoma (LCC) that tends to grow rapidly and spread more aggressively than some other forms of lung cancer. H1581 is characterized by high focal amplification of FGFR1 [61] overexpression, which is related to increased aggressiveness, metastasis, and poor prognosis in various cancer types (especially in NSCLC) [62]. Activation of FGFR1 was reported to initiate EMT in several cancer types, including primary or secondary drug resistant lung cancer and lung cancer cell lines such as H1581 [63]. Importantly, H1581 cell line presents an EMT-derived, highly drug-resistant, cancer stem-cell-like phenotype, with increased ALDH activity. Inhibition of FGFR1 and ALDH activity suppress the growth, viability, and stem-cell-like phenotype of H1581 [64]. Thus, H1581 may be considered a well indicator, partially proving ALDH importance in drug resistance. Supplementation of H1581 with 20 µM cisplatin (24 h) had no significant impact on cell viability; however, the combination of 3 µM **19** and 20 µM cisplatin significantly decreased cell viability, as observed using a standard WST-1 assay (Figure 4F). ITCs 19′s impact on H1581 viability is presented in Supplementary Materials (Figure S2).

### 2.7. Isothiocyanate #19 Decreases Proteolytical Abilities and Invasive Properties of Cisplatin-Resistant NSCLC Cells

**Figure 5:**
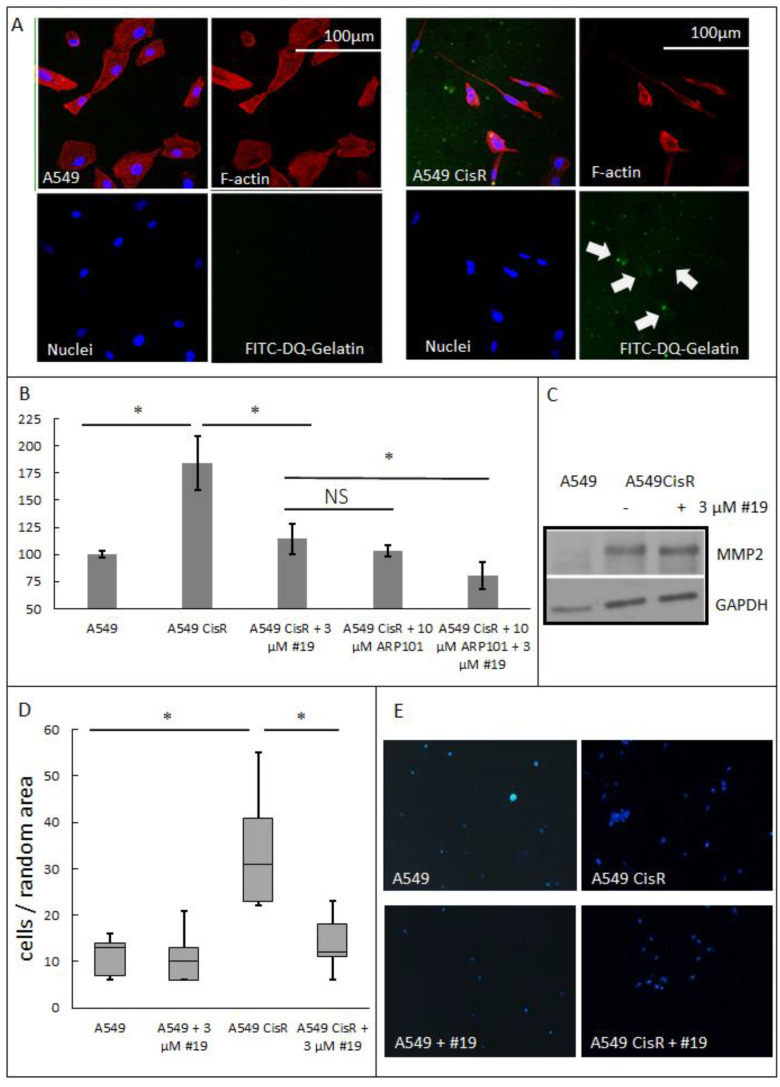
Repression of proteolytical and invasive abilities of A549CisR by isothiocyanates. (**A**) Confocal microscopy image of A549 and A549CisR proteolytic degradation of FITC-conjugated DQ gelatin (Life Technologies, Waltham, MA, USA) (zymography in situ). Cleaved DQ gelatin becomes fluorescently active as highlighted by white arrows. F-actine stained with Texas Red-X Phalloidin, nuclei stained with Hoechst 33342. (**B**) Gelatinolytic activity of A549 and A549CisR calculated by measuring fluorescence intensity of cleaved DQ gelatin. MMP2 inhibitor ARP101 used as an additional control to verify ITCs impact on DG gelatin degradation. Calculated fluorescence intensity in arbitrary units of fluorescence were et as control—100% for A549. * *p* < 0.05, not statistically significant—NS. (**C**) Standard SDS-PAGE Western blot analysis of MMP2 expression in A549, A549CisR, and A549CisR supplementation with compound **19** (24 h 3 µM). (**D**) Gelatin transwell invasion assay based on [57,67]. A549 and A49CisR were treated with or without compound **19** (24 h 3 µM) and transferred to upper chamber in starving medium supplemented with or without **19** 3 µM. Full medium was used as chemoattractant in lower chamber. Invasion was visualized and calculated after 3 h in 5 random spots. * *p* < 0.05. (**E**) Gelatin transwell invasion assay visualization by fluorescence microscopy—random representative image.

The obtained cisplatin-resistant variant of A549 presents a significantly higher migration rate as observed in the wound healing assay. However, migration has two main types: “path finding” (amoeboid migration type) and “path generating” (mesenchymal type of migration). Amoeboid migration is characterized by rounded cell morphology, low adhesion, high migration velocity, extensive cell body deformations caused by actin protrusions or hydrostatic membrane blebs, and its independence of extracellular matrix (ECM) degradation. Thus, amoeboid migration is based on cells’ abilities to find and fit into existing “paths”. On the other hand mesenchymal type of migration is acquired in non-mesenchymal cells via EMT and strongly depends on proteolytic degradation of ECM components (mainly via matrix metalloproteinases—MMPs), which enables crossing of the anatomical boundaries and in-aftermath metastasis [65,66]. Cisplatin resistance is often accompanied by increased metastatic potential. A549CisR presents higher proteolytic abilities than parental A549, as visualized by confocal microscopy imaging (zymography in situ assay—white arrows point increased gelatinolytic effect) (Figure 5A) and calculated using fluorescent dequenching (DQ) gelatin assay (Figure 5B). A549CisR supplemented with 3 µM **19** decreases gelatin degradation to the level observed for parental A549 and A549CisR treated with MMP2 inhibitor ARP101 (24 h, 10 µM). Interestingly, gelatin degradation presented by A549CisR supplemented with both 10 µM ARP101 and 3 µM **19** is slightly, yet statistically significant, lower than the one observed for either of the compounds alone, whereas the MMP2 protein level remains unchanged by **19** (Figure 5C). An increased ability to cleave ECM components such as collagen (or its degraded form—gelatin) is required by cancer cells during invasion and metastasis, as it provides physical disintegration of anatomical boundaries allowing for invasion of surrounding as well as distant metastasis [66]. Thus, invasive properties of A549 and A549CisR were tested (Figure 5D,E). Cells were treated with or without 3 µM **19** for 24 h, and next were transferred to gelatin coated 8 µm pore size upper chamber of Nunc Cell Culture Inserts in starving medium (with or without 3 µM **19**). A full medium was used in lower chamber as chemoattractant. Cells were allowed to enter the membrane pores thru gelatin layer for 3 h. Next, the medium and the gelatin from the top surface of the membrane were removed, the invaded cells on the bottom surface of the membrane were washed 2× with PBS, and then fixed, stained with Hoechst 33342, and counted in five random spots. The cisplatin-resistant variant presents a significantly higher invasion rate than the parental A549 cells. Furthermore, supplementation with **19** significantly decreases invasion of A549CisR, presenting no statistically significant effect on A549.

## 3. Discussion

Currently, the best treatment for most types of carcinomas is surgical excision of the early primary tumor with proper histopathologic margins [68]. However, often due to late diagnosis (advanced stage of tumor), cancer cells are able to increase their mass and invade surrounding/distant tissue, thus preventing surgical removal. In such cases, chemotherapy remains one of the basic treatment modality [5,54]. This fact is extremely important in case of lung cancer: due to the lack of effective early-detection methods, it has one of the highest mortality rates among cancers [1].

Cisplatin is commonly used in many lung cancer types, including squamous cell carcinoma (SCC), large cell carcinoma (LCC), and adenocarcinoma (AC) [69]. Although cisplatin shows remarkable effects during initial treatment, a large majority of patients develop resistance as treatment proceeds, presenting a higher number of secondary tumors after period of remission [5,54]; for example, 30–55% of non-small-cell lung cancer (NSCLC) patients (both adenocarcinoma and squamous cell carcinoma) [70] suffer from cisplatin-resistant cancer reoccurrence within one year of surgery and associated chemotherapy. There are many mechanisms responsible for cisplatin therapy failure such as DNA-damage repair, cell-death inhibition, drugs efflux and inactivation, drug-target alteration, and metabolic shift. However, one of the most prominent mechanisms is EMT induction, which allows an epithelial cell to obtain a mesenchymal phenotype, resulting in an increased ability to migrate [5]. Importantly, cisplatin-related EMT leads to the acquisition of a migratory phenotype, which allows passage across anatomical boundaries and the invasion of both local and distant tissue [71]. Interestingly, inhibition of Ataxia Telangiectasia Mutated (ATM) results in reversion of the EMT phenotype in cisplatin-resistant NSCLC cells, inhibiting cell invasion and tumor metastasis [72]. Although cisplatin resistance is driven by multiple, often ostensibly unrelated mechanisms, the Western blot analysis of our present cisplatin-resistant A549CisR found it to correspond on the protein level with the mRNA profile of a previously described A549CisR variant [73], which is publicly available in the GEO database as the GSE108214 dataset.

Furthermore, in our A549CisR cell line, cisplatin resistance manifests as ongoing EMT, indicated by upregulation of N-cadherin and repression of E-cadherin, which substantially increased migratory potential. Previous studies have also noted a similar co-existence between cisplatin resistance and EMT induction [72,74]. Importantly, our derived A549CisR cell line, representing an EMT phenotype, demonstrated ABCC1 and ABCC4 upregulation and ABCG2 downregulation; this relationship was observed for GSE108214, while ABCG2 repression has also been noted in advanced colorectal cancer cells undergoing EMT and patient samples [57]. Several EMT-inducing transcription factors, such as Twist, Snail, and ZEB, were recently confirmed to be important regulators of certain ABC proteins, directly interacting with E-Box sites in their corresponding promoter regions [7,57]. This mechanism may be utilized by cisplatin-resistant cells to increase overall multidrug resistance, as ABC proteins have broad spectrum of transported chemotherapeutic agents such as 5-fluorouracil, irinotecan, doxorubicin, mitoxantrone, or vinblastine, to name a few [75,76].

Currently, potential supplements to cisplatin-based chemotherapy are being sought [77,78,79]. One such candidate is Disulfiram (DSF), which is an aldehyde dehydrogenase (ADH) inhibitor that has been used as a first-line anti-alcoholism Drug The drug has been reported to cause cell-cycle arrest in the G2/M phase and enhance cisplatin sensitivity in NSCLC lines [80]. Recently, disulfiram has been called “a novel repurposed drug for cancer therapy”, with an anti-cancer effect noted in several cancer types, including liver, breast, prostate, pancreatic, and NSCLC [81]. Therefore, the present study examines the potential of ADH inhibitors for improving the treatment of cisplatin-resistant NSCLC. Moreover, interestingly, aldehyde dehydrogenase (ALDH) is considered to be marker of NSCLC circulating tumor cells (CTC), which indicate an advanced EMT phenotype [82].

Our data confirm that DSF triggers re-sensitization of a cisplatin-resistant NSCLC cell line (A549CisR). Although DSF is known to inhibit NF-kB signaling, proteasome activity, and aldehyde dehydrogenase (ALDH) activity and to induce endoplasmic reticulum (ER) stress and autophagy, the exact mechanisms of its anti-cancer properties remain unclear [81].

Our findings indicate that DSF reverses an acquired mesenchymal phenotype to a certain extent, repressing the expression of mesenchymal marker N-cadherin. Interestingly, DSF is rapidly metabolized to diethyldithiocarbamate (DDTC), which is further converted to *S*-methyl-*N*,*N*-diethyldithiocarbamate (DETC) and *S*-methyl-*N*,*N*-diethyldithiocarbamate (Me-DDTC), and it is these metabolites of DSF that are the true inhibitors of ALDH in vivo [60].

The present study evaluates the potential of de novo synthesized isothiocyanates (ICTs) that resemble DSF. Out of 36 tested ITCs, the three most promising compounds were chosen, viz. **18**, **19**, and **36**: these were found to manifest strong affinity to ALDH isoforms and ABC proteins based on in silico analysis. Interestingly, **18** and **19** have also presented strong anti-tumoral properties in a xenograft zebrafish model [83]. The chosen ITCs appear to be strong anti-cisplatin-resistance agents: they were found to significantly repress cisplatin tolerance in both A549CisR and in the stage 4 NSCLC cell line NCI-H1581 at concentrations 10-fold lower than DSF. ALDH family and/or ABC proteins most probably are not the only important target for ITCs, that are beneficial during anticancer therapy. Benzyl isothiocyanates BITCs are one of the most extensively studied ITCs with regard to cancer chemoprevention, which was proven to inhibit the phosphorylation activities of three major mitogen-activated protein kinases (MAPKs): ERK1/2, p38, and p-JNK1/2 [50].

In a previous study, ITCs were found to demonstrate anti-proliferative activity in vitro in the human colon, uterus, mammary gland, and lung carcinoma cell lines. ITC treatment led to cell-cycle arrest and cell death. ITCs have also been found to be effective against a murine mammary gland carcinoma 4T1 model in vivo, with administration resulting in reduced tumor mass [33]. Furthermore, 3 µM ITC analogs inhibited the motility of three highly malignant cell lines derived from cervical (HeLa), glioblastoma (U87), and breast (MDA-MB-231) carcinomas [83]. This finding is consistent with our present observations.

DSF has been used to treat alcohol abuse for about 70 years. Typically, patients receiving DSF-based therapy are exposed to high doses for a long period of time, i.e., six or more months, during which time the drug appears to possess non-lethal properties, if not mixed with alcohol [84].

In the present study, 15-day supplementation with **19** was found to partially reverse the EMT phenotype (restoration of E-cadherin expression) stem-cell-like phenotype (downregulation of ALDH3A1) of the tested on A549CisR cells, and repress their migratory potential to the level observed in parental A549 cells.

Cell migration is a complicated process consisting of cell-body polarization, followed by the formation and extension of cell protrusions; these protrusions adhere to the substratum, and cell contraction moves the cell body forward toward the leading edge. The migration cycle is completed by deadhesion of the attachments at the rear of the cell [85]. This cascade of events requires substantial energy expense in the form of ATP [86]. Thus, since ALDH mediates the production of NADH, which is used as an energy source for ATP production during oxidative phosphorylation (OXPHOS) [87], long-term ALDH inhibition may slow the migration rate. Importantly, while OXPHOS uses NADH supplied from ALDH in cancer cells, OXPHOS obtains NADH via the tricarboxylic acid cycle (TCA cycle) in normal cells. Thus, targeting cancer cell OXPHOS by inhibiting ALDH could selectively reduce their ATP levels, significantly repressing many of the mechanisms of cancer cells [88]. High ALDH3A1 expression and activity correlates with cell proliferation and resistance against drug toxicity, such as cyclophosphamide, ifosfamide and trofosfamide. Repression of cells proliferation and drug resistance can be observed upon ALDH3A1 directly inhibition by the administration of specific synthetic inhibitors, antisense oligonucleotides, or siRNA [89,90]. Thus, even though ALDH3A1 expression was downregulated in a discrete manner by the tested ITCs in A549CisR cells, inhibition of its activity is the most important factor influencing cell response. Furthermore, mitochondria are able to interact with the nucleus through the retrograde signaling mechanism; this results in the activation of diverse nuclear responses that regulate survival rate, metastasis, and drug resistance [91]. Additionally, mesenchymal type of migration that strongly relies on proteolytical degradation of ECM components is acquired in non-mesenchymal cells via EMT [65,66]. Thus, often cisplatin-resistant cells, presenting EMT phenotype, are characterized by increased proteolytical abilities and a path-generating type of migration [71]. In this study, tested A549CisR presented enhanced gelatinolytic and invasive properties in comparison to the parental A549. Both invasion and gelationlysis were significantly suppressed by 24 h supplementation with 3 µM ITCs **19**. Gelatin is mainly degraded by matrix metalloproteinases 2 and 9 (MMP2 and MMP9), thus, inhibition of their activity results in altered invasion and metastasis. Importantly, **19** in the tested concentration decreases gelatin proteolysis to the level presented by the well-known MMP2 inhibitor ARP101 (10 µM, 24 h), with no changes observed in the MMP2 protein level. Importantly, MMP2 is produced as an inactive proenzyme that requires activation, canonically performed by MMP14 (non-canonically performed by other enzymes, such as MMP2 itself or Cathepsins); thus, the unchanged protein level upon **19** supplementation is less relevant in terms of MMP2 activity and involvement in cell migration [67]. This effect was presented also by benzyl isothiocyanates (BITCs), which was proven to downregulate both MMP2 and MMP9 but, more importantly, to increase the mRNA level of tissue inhibitor of matrix metalloproteinases-2 (TIMP-2) [50]. Thus, we can strongly assume that compound **19** acts as indirect suppressor of MMP2 activity, however, the exact mechanism is yet to be discovered.

Furthermore, in the A549CisR cell line, the ABC protein levels were restored to the parental non-resistant variant: ABCC1 was downregulated whereas ABCG2 was upregulated. Interestingly, neither ABCC1 nor ABCG2 are cisplatin exporters [75,76]; hence, it was unclear why cisplatin resistance regulates ABC protein levels, while not being a substrate for particular transporters itself, and why compound **19** reverses their level to the one observed in parental A549 cells [92]. However, cisplatin-resistant cells exhibit an EMT phenotype, which has been proposed to be the main cause of the primary and acquired drug resistance in several cancer types [7,93]. The observed downregulation of ABCG2 in the more mesenchymal A549CisR cell line may be difficult to explain, but our data correspond well with previous findings, indicating that the downregulation of ABCG2, at both the mRNA and protein level, reverses the correlation with mesenchymal markers and acquisition of advance EMT in CRC cells (in vivo and in vitro) [57]. Furthermore, long-term supplementation with compound **19** partially represses and reverses EMT, i.e., restores E-Cadherin expression, and EMT triggers the metastatic and drug-resistance properties of cisplatin-resistant NSCLC cells.

Importantly, compound **19** presents high affinity to ABC proteins, including ABCB1, ABCC1, and ABCG2, which are present in various pharmaceuticals, such as Doxorubicin, Paclitaxel, Vinblastine, Methotreaxate (MTX), Irinotecan, and Topotecan [75,76]. This can potentially increase their cytosolic accumulation, leading to the repression of cisplatin-induced multidrug resistance and the resensitization of cells; however, this needs further investigation. Compound **19** appears to possess strong anti-cisplatin/anti-multidrug resistance and anti-metastatic properties, and its supplementation during chemotherapy may be highly beneficial for NSCLC patients.

## 4. Materials and Methods

### 4.1. Cell Culturing and Induction of Drug Resistance

Lung cancer cell lines A549 and NCI-H1581 were obtained from the ATCC (Manassas, VA, USA). The A549 cisplatin-resistant sub-line A549CisR was established by growing A549 cells in the presence of increasing concentrations of cisplatin to a final concentration of 10μM over approximately six months. Both cell lines (A549/A549CisR) were cultured in Ham’s F12-K medium (Corning, Manassas, VA, USA), H1581 were cultured in DNEM/HAM F-12 (Corning, Manassas, VA, USA) in a 90–95% humidified atmosphere of 5% CO_2_; the media were supplemented with 10% heat-inactivated fetal bovine serum (FBS) (Biowest, Nuaillé, France) and the antibiotics streptomycin, penicillin (Biowest), and primocin (Invivogen, San Diego, CA, USA). The cells were plated in 25 cm^2^ cell culture flasks and sub-cultured before reaching confluency using Accutase (Biowest). The culture medium was changed every two days. The cells were split 1:10 during each passage.

### 4.2. Reagents

Cisplatin [cis-diammineplatinum(II) dichloride], was obtained from Sigma-Aldrich (St. Louis, MO, USA) and dissolved in DMSO. Disulfiram was obtained from Cayman Chemical and dissolved in DMSO. Aliquots were stored at −20 °C for up to a maximum of three months and thawed immediately before use. WST-1 [2-(4-Iodophenyl)-3-(4-nitrophenyl)-5-(2,4-disulfophenyl)-2H-tetrazolium] was purchased from ScienCell (Research Lab., Carlsbad, CA, USA) freshly made, #8038; RIPA lysis buffer was purchased from VWR Chemicals, #N653-100 mL; protease inhibitor was purchased from Thermo Fisher Scientific (Waltham, MA, USA), #PIER87785; BCA purchased from Thermo Fisher Scientific, #PIER23225. Primary antibodies: E cadherin #3195P cell signaling, N cadherin #13116P cell signaling, ABCC1 #VMA00330 BioRad (Hercules, CA, USA), ABCC4 #PA5-18315 Thermo Fisher Scientific, ABCG2 #BRB155559 Biorbyt (Cambridge, UK), GAPDH #sc-32233 Santa Cruz Biotechnology (Dallas, TX, USA), α-Tubulin #NB100-690H Novus Biologicals (Littleton, CO, USA), ALDH3A1 #PA5-80332 Thermo Fisher Scientific, ALDH7A1 #MA5-29028 Thermo Fisher Scientific. Secondary HRP-conjugated antibodies were purchased from Santa Cruz Biotechnology.

### 4.3. Western Immunoblotting

Total protein was extracted from cells using ice-cold M-PER Mammalian Protein Extraction Reagent #78501 supplemented with the Halt protease inhibitor cocktail (Thermo Scientific, Waltham, MA, USA), and the soluble protein fraction was collected through centrifugation. The protein concentrations in the cell lysates were measured with the BCA method (Pierce/Thermo Scientific, Waltham, MA, USA) and equalized between samples. Protein (40 µg) from whole cell lysates was fractionated on SDS-PAGE gels and transferred to a PVDF or nitrocellulose membrane (BioRad, Hercules, CA, USA). Transfer efficiency and loading were confirmed by reversible staining of the membrane with Ponseau S solution (Sigma-Aldrich, UK) following protein transfer. Membranes were blocked at room temperature with blocking buffer (BioRad, Hercules, CA, USA). Primary antibodies were added in 1:1000–1:5000 dilution and incubated for one hour at RT (Materials and Methods Section 4.2). Membranes were washed 3 × 15′ with TBST and incubated with a secondary horseradish peroxidase (HRP)—labelled antibody for 1 h RT (1:2000). Membranes were washed in 3 × 15′ with TBST following incubation with secondary antibodies. Bound antibody complexes were detected and visualized using Clarity Western ECL Substrate (BioRad, Hercules, CA, USA). Densitometric analysis was carried out using ImageJ software, and percentage expression was represented relative to controls (100%) [94].

### 4.4. Cell Viability Assay

The in vitro cell viability effects of DSF, analogs of disulfiram, cisplatin, DSF/cisplatin, and analogs of disulfiram/cisplatin were determined by WST-1 assay. In brief, the cells (2 × 10^5^ cells/mL) were seeded on 96-well culture plates and left for 24 h. Next, parental and resistant tumor cells were incubated in 100 µL of fresh medium containing different drug concentrations. Next, after 48 h of incubation, 10 µL of WST-1 reagent (ScienCell, Research Lab., Carlsbad, CA, USA), freshly made, #8038, was added for 2 h. Calculation of cell viability was done by OD_450 nm_–OD_630 nm_ using the BioTek ELx800 multimode microplate reader.

### 4.5. Wound Healing (Scratch) Assay

The 2D migration was tested using wound healing assay. Treated or untreated with ITC **19** (15 days, 3 µM), A549 and A549CisR cells were seeded on a six-well plate and grown to confluence. Wounds were created by scraping monolayer cells using a 20 μL pipet tip, and non-adherent cells were rinsed off twice with PBS. Fresh medium was added with or without 3 µM ITC **19**. The scratch and surrounding cells were imaged at 0 h (immediately after scratching). The area of the wound was visualized (OLYMPUS IX53 microscope, magnification, 100×) and measured 4, 8, 10, and 24 h after scratching. The wound area was calculated by ImageJ software. Cell motility was estimated by quantification of percentage recovery using the equation: R (%) = [1 − (wound area at Tt/wound area at T0)] × 100, where T0 is the wounded area at 0 h, and Tt is the wounded area after th. The assays were replicated three times.

### 4.6. GEO Database Analysis

Microarray profiles and the GSE108214 dataset of the NSCLC cell line A549 and its cisplatin-resistant variant (A549CisR) were acquired from the public Gene Expression Omnibus (GEO) databases—National Center for Biotechnology Information (NCBI), USA National Library of Medicine 8600 Rockville Pike, Bethesda, MD 20894, USA (https://www.ncbi.nlm.nih.gov/geo/, accessed on 14 April 2021) [59]; these were described previously [5]. The data were analyzed and presented as box charts, with the median depicted, as described previously [57]. Statistical analysis was performed using Jasp software (https://jasp-stats.org/, accessed on 6 May 2022) [95].

### 4.7. Synthesis of ITCs—Similar Compounds to DSF

**Scheme 1:**
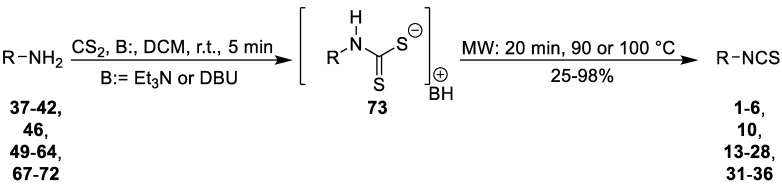
Method A: microwave-assisted synthesis of isothiocyanates **1**–**6**, **10**, **13**–**28**, and **31**–**36**.

**Scheme 2:**
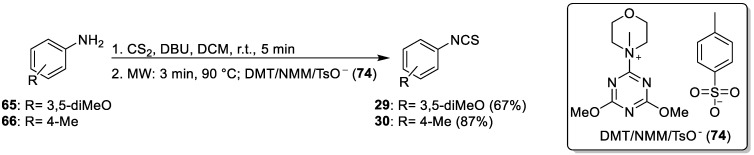
Method B: microwave-assisted synthesis of isothiocyanates **29** and **30** using DMT/NMM/TsO^−^ (**74**) as desulfurating reagent.

**Scheme 3:**
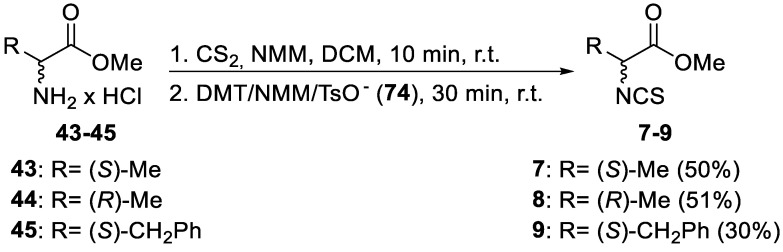
Method C: synthesis of isothiocyanate derivatives of methyl ester amino acids **7**–**9** using DMT/NMM/TsO^−^ (**74**) as desulfurating reagent.

**Scheme 4:**
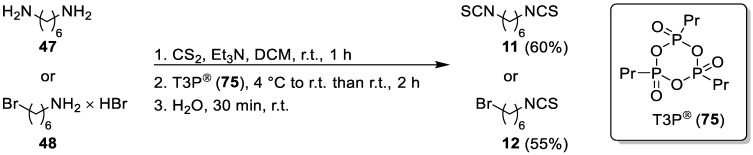
Method D: synthesis of isothiocyanates **11** and **12** using propane phosphonic acid anhydride (T3P) (**75**).

The tested isothiocyanates **1**–**36** (Table 1) had previously been synthesized using four known methods [96,97,98] (Scheme 1, Scheme 2, Scheme 3 and Scheme 4, Methods A–D).

The isothiocyanates **1**–**6**, **10**, **13**–**28**, and **31**–**36** were obtained by one-pot, two-step, microwave-assisted (MW) synthesis (Method A). Briefly, a mixture of aliphatic primary amines **37**–**42**, **46**, and **49**–**55** or aromatic amines **56**–**64** and **67**–**72**, carbon disulfide (CS_2_) and triethylamine (Et_3_N) (for amines **37**–**42**, **46**, and **49**–**55**), or DBU (for amines **56**–**64** and **67**–**72**) were transformed under normal conditions at room temperature into the intermediate dithiocarbamates **73**. Next, dithiocarbamates **73** were converted without any desulfurating reagent under microwave-assisted reaction (MW: 20 min, 90 °C for **37**–**42**, **46**, and **49**–**55** or 100 °C for **56**–**64** and **67**–**72**) to ITCs **1**–**6**, **10**, **13**–**28**, and **31**–**36** in good yields (25–98%) (Scheme 1, Table 1) [96].

Isothiocyanates **29**–**30** were also synthesized by one-pot, two-step microwave-assisted synthesis using CS_2_, DBU, and aromatic amines **65** and **66** as substrates; however, a different MW method was used (Method B). The intermediate dithiocarbamates were synthesized, as shown in Method A. The second step however, performed under microwave-assisted conditions, was accomplished in a shorter time (MW: 3 min and 90 °C) and in the presence of 4-(4,6-dimethoxy-1,3,5-triazin-2-yl)-4-methylmorpholinium toluene-4-sulfonate (DMT/NMM/TsO^−^, **74**) as a desulfurating reagent. Isothiocyanates **29** and **30** were isolated at high yields (67–87%) (Scheme 2) (Table 1) [97].

Isothiocyanate derivatives of methyl ester amino acids **7**–**9** were obtained under normal conditions in a one-pot, two-step procedure in the presence of DMT/NMM/TsO^−^ (**74**) as desulfurating agent; however, the reaction was performed in normal conditions (Method C). The first step was performed in the presence of CS_2_, NMM at room temperature in 10 min, using hydrochloride **43**–**45** as substrates; the second step was performed with desulfurating reagent **74** at room temperature and in 30 min. ITCs **7**–**9** were isolated with satisfactory yields (30–51%) after flash chromatography (Scheme 3) (Table 1) [97].

The last two isothiocyanates **11** and **12** were synthesized in a two-step process with propane phosphonic acid anhydride (T3P) used as a desulfurating reagent (**75**) (Method D). Diamine **47** or hydrobromide **48** was reacted with CS_2_ in the presence of Et_3_N at room temperature for one hour. Next, the reactions were cooled to 4 °C, T3P (**75**) was added in two portions, and the mixture was stirred for another two hours at room temperature. Products **11** and **12** were isolated in good yields (55–60%) using flash chromatography (Scheme 4) (Table 1) [98].

### 4.8. Molecular Modeling

Affinity calculations were performed for 26 ABC proteins and 27 ligands using software dedicated to molecular docking: AutoDockTools v.1.5.6 (La Jolla, California, USA), included in the MGLTools 1.5.6 (La Jolla, California, USA) package, and AutoDock Vina 1.1.2 (La Jolla, California, USA). Files describing proteins were downloaded in pdb format from the RCSB Protein Data Bank [99] and then converted using AutoDockTools to the pdbqt format required by AutoDock Vina.

The structures of ITCs **1**–**37** were drawn in ACD/ChemSketch (Toronto, Ontario, Canada) (Freeware) and then 3D Structure Optimization was performed. The structures were saved in .mol (MDL MOL) format. Next, the structures in .mol format were converted to .pdb (Protein Data Bank) format using OpenBabelGUI. The .pdb format files were used to model docking in the Vina package.

AutoDockTools was also used to determine the size of the search area and its center. For protein molecules with size that exceeded the maximum size of the search area, search sub-areas totaling the entire protein molecule were defined. For example, two search sub-areas covering the entire molecule were created for the 6qex protein, and these were treated as separate cases in the calculations: 6qex(1) and 6qex(2).

**Figure 6:**
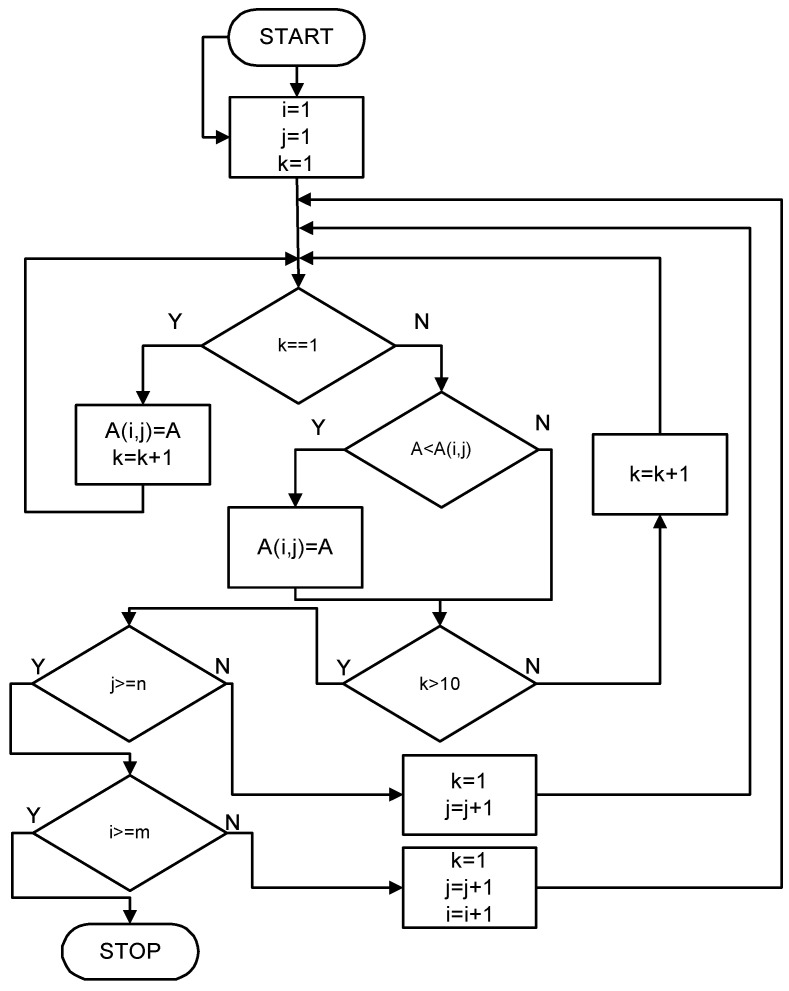
Flow diagram of the algorithm controlling the calculations, where i—protein index; j—ligand index; k—search variant index; m—number of proteins; n—number of ligands; A—affinity for the current protein and ligand; A_set_—set of affinity values.

As a non-deterministic search algorithm was implemented in AutoDock Vina, each docking variant was calculated 10-fold. As a result, it was necessary to perform 9620 calculations (37 ligands × 26 proteins × 10 searches). As it would be difficult to run AutoDock Vina manually so many times, the calculation process was automated using a script written in Matlab 2021a. This script, in addition to running individual calculation cases, aggregated the results generated by AutoDock Vina. The calculation process is given in the flow diagram presented in Figure 6.

The calculations were carried out on a PC equipped with a 12-core/24-thread AMD Ryzen 9 3900X processor. In order to fully utilize all processor cores, three instances of AutoDock Vina were run simultaneously. In order to make a quantitative assessment of the affinity of the studied ligands to selected proteins, the average affinity values of the analyzed ligands for selected proteins, expressed by the relationship (1), were calculated:

(1) $$AA\left(j\right)=\frac{\sum_{i=1}^{i=m}A\left(i,j\right)}{m}$$

where *i*—protein index; *j*—ligand index; k—search variant index; *m*—number of proteins; n—number of ligands; *A*—affinity; *AA*—average affinity.

### 4.9. Fluorescent Dequenching (DQ) Gelatin Assay

The surface of 96-well plates was coated with 75 µL 0.1 mg/mL DQ gelatin (Life Technologies, Waltham, MA, USA) overnight at 4 °C and then washed 3× with PBS. Then, 25 × 10^5^ cells/mL were added for 24 h to earlier prepared DQ gelatin-coated dishes in full medium. Additionally, as a control medium supplemented with 10 µM MMP-2 inhibitor ARP101 was used. FITC fluorescence generated by the cleavage of DQ gelatin was measured using a Thermo Labsystem Fluoroscan Ascent reader (ThermoFisher Scientific, Waltham, MA, USA), fit with FITC excitation and emission filters. Data are presented as the percent of increase above background fluorescence (100%) observed in the control A549 cell line [67].

Visualization of DQ gelatin assay (zymography in situ assay) was performed as described by us in [67]. Briefly, cells were grown on FITC gelatine-coated chamber slides (Life Technologies, Waltham, MA, USA) until 60–70% confluency and were subsequently incubated with Hoechst 33342 (Molecular Probes/Life Technologies, Waltham, MA, USA) for 15 min in the incubator. The cells were washed with PBS (3×), fixed for 10 min in CellFIX^TM^ (1% formaldehyde, 0.35% methanol, 0.09% sodium azide) from BD Biosciences (cat no. 340181) for 10 min, washed with PBS (3 × 5 min), and blocked with 3% BSA/PBS at RT for 1 h. After washing with PBS, the slides were incubated with F-actin probe—Texas Red-X Phalloidin (ThermoFisher Scientific # T7471) at RT for 20 min in the dark. The slides were washed with PBS and mounted with Mowiol (Sigma-Aldrich, St. Louis, MO, USA), and the cells were visualized under a confocal microscope (Nikon D-Eclipse C1; Nikon, Tokyo, Japan) with a 40× objective and were analyzed with EZ-C1 version 3.6 software (Tokyo, Japan).

### 4.10. Transwell Invasion Assay

Transwell invasion assay was performed using our modified protocol [57,67]. Briefly: Nunc Cell Culture Inserts (transwell) with 8.0μm pore diameter (#141006) were covered with 50 µL 0.2% gelatin 1 h 37 °C. Next, gelatin was carefully removed. A549 or A549CisR cells were treated with or without 3 µM #19 for 24 h. Then, they were trypsinized, washed 2× with medium, and transferred (2 × 10^5^ cells/chamber) to upper chamber of Nunc Cell Culture Inserts in 0.1% BSA medium—supplemented with or without 3 µM #19. Full medium in lower chamber was used as chemoattractant. Next, medium and the gelatin from the top surface of the membrane were removed, invaded cells on the bottom surface of the membrane were washed 2× with PBS and then fixed for 5 min with CellFIX^TM^. Cells were dyed at RT 15 min with Hoechst 33,342 (Molecular Probes/Life Technologies, Waltham, MA, USA). Finally, membranes were cut out from chambers and placed on microscope glass, and number of cells that migrate into the membrane was counted in 5 random spots.

## 5. Conclusions

The presence of significant drug resistance, as well as the combination of cisplatin resistance and increased metastatic potential in the case of NSCLC, remains an obstacle during anticancer therapy. Chemotherapy can result in the selection of highly resistant and metastatic cancer cells, and this may be one of the reasons why patients present a high number of secondary tumors after apparent post-chemotherapeutic reemission [54]. Thus, long-term therapeutic strategies should focus on adjuvant “adaptive therapies” before resistance emerges [100]. Our research indicates that DSF and the tested ITCs have no impact on NSCLC cell viability at the tested concentrations, but significantly repress the cisplatin resistance of both cisplatin-resistant and metastatic NSCLC cells. Furthermore, supplementation with the tested ITCs significantly decreased the metastatic potential of all tested NSCLC models, reversing EMT toward an epithelial phenotype and decreasing the migratory potential as well as the proteolytic and invasive abilities. Therefore, the tested ITCs, especially ITC **19**, possess anti-drug-resistant and anti-metastatic potential, which may be of value during cisplatin-based chemotherapy.

## Figures and Tables

**Table 1:** Structure of tested isothiocyanates **1**–**36** ^a^.

Substrate		Isothiocyanate (Product)		Yield %	Method
Symbol	Structure	Symbol	Structure		
**37**	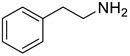	**1**	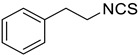	87	A
**38**	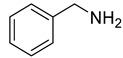	**2**	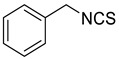	83	A
**39**	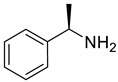	**3**	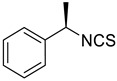	83	A
**40**	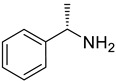	**4**	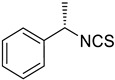	85	A
**41**	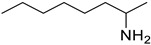	**5**	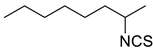	83	A
**42**	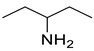	**6**	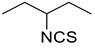	76	A
**43**	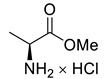	**7**	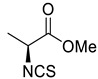	50	C
**44**	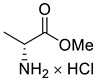	**8**	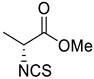	51	C
**45**	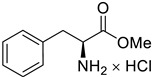	**9**	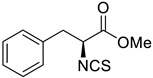	30	C
**46**	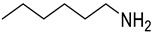	**10**	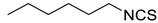	82	A
**47**	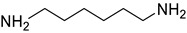	**11**	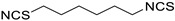	60	D
**48**	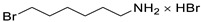	**12**	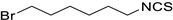	55	D
**49**	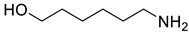	**13**	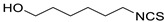	75	A
**50**	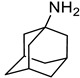	**14**	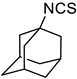	82	A
**51**	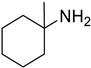	**15**	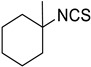	54	A
**52**	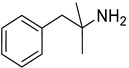	**16**	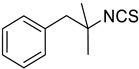	82	A
**53**	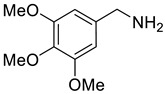	**17**	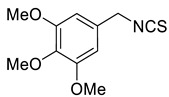	53	A
**54**	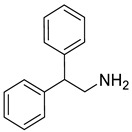	**18**	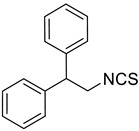	79	A
**55**	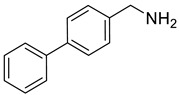	**19**	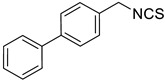	61	A
**56**	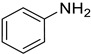	**20**	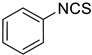	77	A
**57**	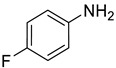	**21**	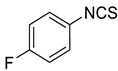	61	A
**58**	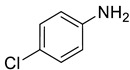	**22**	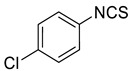	75	A
**59**	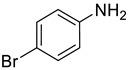	**23**	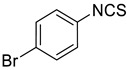	95	A
**60**	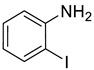	**24**	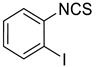	54	A
**61**	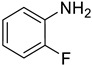	**25**	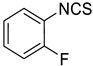	62	A
**62**	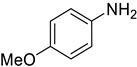	**26**	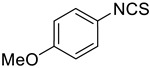	75	A
**63**	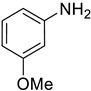	**27**	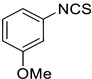	80	A
**64**	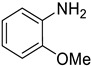	**28**	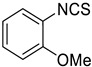	87	A
**65**	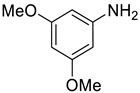	**29**	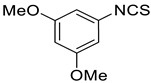	67	B
**66**	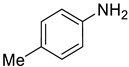	**30**	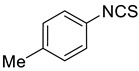	87	B
**67**	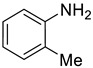	**31**	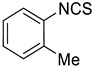	90	A
**68**	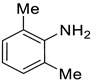	**32**	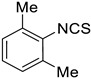	98	A
**69**	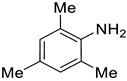	**33**	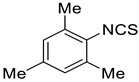	94	A
**70**	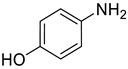	**34**	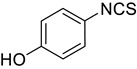	72	A
**71**	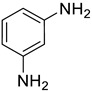	**35**	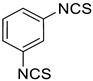	57	A
**72**	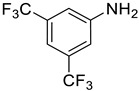	**36**	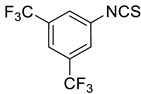	25	A

^a^ Method A [96]; method B and method C [97]; method D [98].

## Data Availability

Gene Expression Omnibus dataset #GSE108214 is available at https://www.ncbi.nlm.nih.gov/geo/query/acc.cgi?acc=GSE108214, accessed on 21 July 2022.

## Supplementary Materials

The supporting information can be downloaded at: https://www.mdpi.com/article/10.3390/ijms23158644/s1.

## References

[1] Kryczka J., Migdalska-Sęk M., Kordiak J., Kiszałkiewicz J.M., Pastuszak-Lewandoska D., Antczak A., Brzeziańska-Lasota E. (2021). Serum Extracellular Vesicle-Derived MiRNAs in Patients with Non-Small Cell Lung Cancer-Search for Non-Invasive Diagnostic Biomarkers. Diagnostics.

[2] Cancer Today. http://gco.iarc.fr/today/home.

[3] Lung Cancer—Non-Small Cell—Statistics. https://www.cancer.net/cancer-types/lung-cancer-non-small-cell/statistics.

[4] MacDonagh L., Gallagher M.F., Ffrench B., Gasch C., Breen E., Gray S.G., Nicholson S., Leonard N., Ryan R., Young V. (2017). Targeting the Cancer Stem Cell Marker, Aldehyde Dehydrogenase 1, to Circumvent Cisplatin Resistance in NSCLC. Oncotarget.

[5] Kryczka J., Kryczka J., Czarnecka-Chrebelska K.H., Brzeziańska-Lasota E. (2021). Molecular Mechanisms of Chemoresistance Induced by Cisplatin in NSCLC Cancer Therapy. Int. J. Mol. Sci..

[6] Sobczak M., Strachowska M., Gronkowska K., Robaszkiewicz A. (2022). Activation of ABCC Genes by Cisplatin Depends on the CoREST Occurrence at Their Promoters in A549 and MDA-MB-231 Cell Lines. Cancers.

[7] Jiang Z.-S., Sun Y.-Z., Wang S.-M., Ruan J.-S. (2017). Epithelial-Mesenchymal Transition: Potential Regulator of ABC Transporters in Tumor Progression. J. Cancer.

[8] Januchowski R., Wojtowicz K., Zabel M. (2013). The Role of Aldehyde Dehydrogenase (ALDH) in Cancer Drug Resistance. Biomed. Pharmacother..

[9] Kang J.H., Lee S.-H., Lee J.-S., Nam B., Seong T.W., Son J., Jang H., Hong K.M., Lee C., Kim S.-Y. (2016). Aldehyde Dehydrogenase Inhibition Combined with Phenformin Treatment Reversed NSCLC through ATP Depletion. Oncotarget.

[10] Tomita H., Tanaka K., Tanaka T., Hara A. (2016). Aldehyde Dehydrogenase 1A1 in Stem Cells and Cancer. Oncotarget.

[11] Wang N.-N., Wang L.-H., Li Y., Fu S.-Y., Xue X., Jia L.-N., Yuan X.-Z., Wang Y.-T., Tang X., Yang J.-Y. (2018). Targeting ALDH2 with Disulfiram/Copper Reverses the Resistance of Cancer Cells to Microtubule Inhibitors. Exp. Cell Res..

[12] Yokoyama A., Muramatsu T., Omori T., Yokoyama T., Matsushita S., Higuchi S., Maruyama K., Ishii H. (2001). Alcohol and Aldehyde Dehydrogenase Gene Polymorphisms and Oropharyngolaryngeal, Esophageal and Stomach Cancers in Japanese Alcoholics. Carcinogenesis.

[13] Marchitti S.A., Brocker C., Stagos D., Vasiliou V. (2008). Non-P450 Aldehyde Oxidizing Enzymes: The Aldehyde Dehydrogenase Superfamily. Expert Opin. Drug Metab. Toxicol..

[14] Bell L., Oloyede O.O., Lignou S., Wagstaff C., Methven L. (2018). Taste and Flavor Perceptions of Glucosinolates, Isothiocyanates, and Related Compounds. Mol. Nutr. Food Res..

[15] Avato P., Argentieri M.P. (2015). Brassicaceae: A Rich Source of Health Improving Phytochemicals. Phytochem. Rev..

[16] Verhoeven D.T., Verhagen H., Goldbohm R.A., van den Brandt P.A., van Poppel G. (1997). A Review of Mechanisms Underlying Anticarcinogenicity by Brassica Vegetables. Chem. Biol. Interact..

[17] Rollin P., Tatibouët A. (2011). Glucosinolates: The Synthetic Approach. C. R. Chim..

[18] Bell L., Wagstaff C. (2014). Glucosinolates, Myrosinase Hydrolysis Products, and Flavonols Found in Rocket (Eruca Sativa and Diplotaxis Tenuifolia). J. Agric. Food Chem..

[19] Soundararajan P., Kim J.S. (2018). Anti-Carcinogenic Glucosinolates in Cruciferous Vegetables and Their Antagonistic Effects on Prevention of Cancers. Molecules.

[20] Maina S., Misinzo G., Bakari G., Kim H.-Y. (2020). Human, Animal and Plant Health Benefits of Glucosinolates and Strategies for Enhanced Bioactivity: A Systematic Review. Molecules.

[21] Rao C.V. (2013). Benzyl Isothiocyanate: Double Trouble for Breast Cancer Cells. Cancer Prev. Res..

[22] Dinh T.N., Parat M.-O., Ong Y.S., Khaw K.Y. (2021). Anticancer Activities of Dietary Benzyl Isothiocyanate: A Comprehensive Review. Pharmacol. Res..

[23] Coscueta E.R., Sousa A.S., Reis C.A., Pintado M.M. (2022). Phenylethyl Isothiocyanate: A Bioactive Agent for Gastrointestinal Health. Molecules.

[24] Qin C.-Z., Zhang X., Wu L.-X., Wen C.-J., Hu L., Lv Q.-L., Shen D.-Y., Zhou H.-H. (2015). Advances in Molecular Signaling Mechanisms of β-Phenethyl Isothiocyanate Antitumor Effects. J. Agric. Food Chem..

[25] Briones-Herrera A., Eugenio-Pérez D., Reyes-Ocampo J.G., Rivera-Mancía S., Pedraza-Chaverri J. (2018). New Highlights on the Health-Improving Effects of Sulforaphane. Food Funct..

[26] Jiang X., Liu Y., Ma L., Ji R., Qu Y., Xin Y., Lv G. (2018). Chemopreventive Activity of Sulforaphane. Drug Des. Devel. Ther..

[27] Leone A., Diorio G., Sexton W., Schell M., Alexandrow M., Fahey J.W., Kumar N.B. (2017). Sulforaphane for the Chemoprevention of Bladder Cancer: Molecular Mechanism Targeted Approach. Oncotarget.

[28] Khan S., Awan K.A., Iqbal M.J. (2022). Sulforaphane as a Potential Remedy against Cancer: Comprehensive Mechanistic Review. J. Food Biochem..

[29] Kaiser A.E., Baniasadi M., Giansiracusa D., Giansiracusa M., Garcia M., Fryda Z., Wong T.L., Bishayee A. (2021). Sulforaphane: A Broccoli Bioactive Phytocompound with Cancer Preventive Potential. Cancers.

[30] Mahn A., Castillo A. (2021). Potential of Sulforaphane as a Natural Immune System Enhancer: A Review. Molecules.

[31] Kiełbasiński P., Łuczak J., Cierpiał T., Błaszczyk J., Sieroń L., Wiktorska K., Lubelska K., Milczarek M., Chilmończyk Z. (2014). New Enantiomeric Fluorine-Containing Derivatives of Sulforaphane: Synthesis, Absolute Configurations and Biological Activity. Eur. J. Med. Chem..

[32] Cierpiał T., Kiełbasiński P., Kwiatkowska M., Łyżwa P., Lubelska K., Kuran D., Dąbrowska A., Kruszewska H., Mielczarek L., Chilmonczyk Z. (2020). Fluoroaryl Analogs of Sulforaphane—A Group of Compounds of Anticancer and Antimicrobial Activity. Bioorg. Chem..

[33] Psurski M., Janczewski Ł., Świtalska M., Gajda A., Goszczyński T.M., Oleksyszyn J., Wietrzyk J., Gajda T. (2017). Novel Phosphonate Analogs of Sulforaphane: Synthesis, in Vitro and in Vivo Anticancer Activity. Eur. J. Med. Chem..

[34] Gajda T., Janczewski Ł., Psurski M., Świtalska M., Gajda A., Goszczyński T., Oleksyszyn J., Wietrzyk J. (2017). Design, Synthesis, and Evaluation of ω-(Isothiocyanato)Alkylphosphinates and Phosphine Oxides as Antiproliferative Agents. ChemMedChem.

[35] Janczewski Ł. (2022). Sulforaphane and Its Bifunctional Analogs: Synthesis and Biological Activity. Molecules.

[36] Pace V., Monticelli S., de la Vega-Hernández K., Castoldi L. (2016). Isocyanates and Isothiocyanates as Versatile Platforms for Accessing (Thio)Amide-Type Compounds. Org. Biomol. Chem..

[37] Milelli A., Fimognari C., Ticchi N., Neviani P., Minarini A., Tumiatti V. (2014). Isothiocyanate Synthetic Analogs: Biological Activities, Structure-Activity Relationships and Synthetic Strategies. Mini. Rev. Med. Chem..

[38] Gründemann C., Huber R. (2018). Chemoprevention with Isothiocyanates—From Bench to Bedside. Cancer Lett..

[39] Mastuo T., Miyata Y., Yuno T., Mukae Y., Otsubo A., Mitsunari K., Ohba K., Sakai H. (2020). Molecular Mechanisms of the Anti-Cancer Effects of Isothiocyanates from Cruciferous Vegetables in Bladder Cancer. Molecules.

[40] Mitsiogianni M., Koutsidis G., Mavroudis N., Trafalis D.T., Botaitis S., Franco R., Zoumpourlis V., Amery T., Galanis A., Pappa A. (2019). The Role of Isothiocyanates as Cancer Chemo-Preventive, Chemo-Therapeutic and Anti-Melanoma Agents. Antioxidants.

[41] Palliyaguru D.L., Yuan J.-M., Kensler T.W., Fahey J.W. (2018). Isothiocyanates: Translating the Power of Plants to People. Mol. Nutr. Food Res..

[42] Dufour V., Stahl M., Baysse C. (2015). The Antibacterial Properties of Isothiocyanates. Microbiology.

[43] Romeo L., Iori R., Rollin P., Bramanti P., Mazzon E. (2018). Isothiocyanates: An Overview of Their Antimicrobial Activity against Human Infections. Molecules.

[44] Janczewski Ł., Burchacka E., Psurski M., Ciekot J., Gajda A., Gajda T. (2019). New Diaryl ω-(Isothiocyanato)Alkylphosphonates and Their Mercapturic Acids as Potential Antibacterial Agents. Life Sci..

[45] Myzak M.C., Hardin K., Wang R., Dashwood R.H., Ho E. (2006). Sulforaphane Inhibits Histone Deacetylase Activity in BPH-1, LnCaP and PC-3 Prostate Epithelial Cells. Carcinogenesis.

[46] Choi S., Lew K.L., Xiao H., Herman-Antosiewicz A., Xiao D., Brown C.K., Singh S.V. (2007). D,L-Sulforaphane-Induced Cell Death in Human Prostate Cancer Cells Is Regulated by Inhibitor of Apoptosis Family Proteins and Apaf-1. Carcinogenesis.

[47] Myzak M.C., Karplus P.A., Chung F.-L., Dashwood R.H. (2004). A Novel Mechanism of Chemoprotection by Sulforaphane: Inhibition of Histone Deacetylase. Cancer Res..

[48] Asakage M., Tsuno N.H., Kitayama J., Tsuchiya T., Yoneyama S., Yamada J., Okaji Y., Kaisaki S., Osada T., Takahashi K. (2006). Sulforaphane Induces Inhibition of Human Umbilical Vein Endothelial Cells Proliferation by Apoptosis. Angiogenesis.

[49] Thejass P., Kuttan G. (2006). Antimetastatic Activity of Sulforaphane. Life Sci..

[50] Hwang E.-S., Lee H.J. (2008). Benzyl Isothiocyanate Inhibits Metalloproteinase-2/-9 Expression by Suppressing the Mitogen-Activated Protein Kinase in SK-Hep1 Human Hepatoma Cells. Food Chem. Toxicol..

[51] Yu M., Zhang C., Li L., Dong S., Zhang N., Tong X. (2014). Cx43 Reverses the Resistance of A549 Lung Adenocarcinoma Cells to Cisplatin by Inhibiting EMT. Oncol. Rep..

[52] Zhao Z., Zhang L., Yao Q., Tao Z. (2015). MiR-15b Regulates Cisplatin Resistance and Metastasis by Targeting PEBP4 in Human Lung Adenocarcinoma Cells. Cancer Gene. Ther..

[53] Sinha D., Saha P., Samanta A., Bishayee A. (2020). Emerging Concepts of Hybrid Epithelial-to-Mesenchymal Transition in Cancer Progression. Biomolecules.

[54] Kryczka J., Boncela J. (2018). Cell Migration Related to MDR—Another Impediment to Effective Chemotherapy?. Molecules.

[55] Lundholm L., Hååg P., Zong D., Juntti T., Mörk B., Lewensohn R., Viktorsson K. (2013). Resistance to DNA-Damaging Treatment in Non-Small Cell Lung Cancer Tumor-Initiating Cells Involves Reduced DNA-PK/ATM Activation and Diminished Cell Cycle Arrest. Cell. Death Dis..

[56] Yang Z., Guo F., Albers A.E., Sehouli J., Kaufmann A.M. (2019). Disulfiram Modulates ROS Accumulation and Overcomes Synergistically Cisplatin Resistance in Breast Cancer Cell Lines. Biomed. Pharmacother..

[57] Kryczka J., Sochacka E., Papiewska-Pająk I., Boncela J. (2020). Implications of ABCC4–Mediated CAMP Efflux for CRC Migration. Cancers.

[58] Saxena M., Stephens M.A., Pathak H., Rangarajan A. (2011). Transcription Factors That Mediate Epithelial–Mesenchymal Transition Lead to Multidrug Resistance by Upregulating ABC Transporters. Cell. Death Dis..

[59] Home—GEO—NCBI. https://www.ncbi.nlm.nih.gov/geo/.

[60] Skrott Z., Majera D., Gursky J., Buchtova T., Hajduch M., Mistrik M., Bartek J. (2019). Disulfiram’s Anti-Cancer Activity Reflects Targeting NPL4, Not Inhibition of Aldehyde Dehydrogenase. Oncogene.

[61] Fumarola C., Bozza N., Castelli R., Ferlenghi F., Marseglia G., Lodola A., Bonelli M., La Monica S., Cretella D., Alfieri R. (2019). Expanding the Arsenal of FGFR Inhibitors: A Novel Chloroacetamide Derivative as a New Irreversible Agent with Anti-Proliferative Activity Against FGFR1-Amplified Lung Cancer Cell Lines. Front. Oncol..

[62] Deng C., Xiong J., Gu X., Chen X., Wu S., Wang Z., Wang D., Tu J., Xie J. (2017). Novel Recombinant Immunotoxin of EGFR Specific Nanobody Fused with Cucurmosin, Construction and Antitumor Efficiency in Vitro. Oncotarget.

[63] Wang K., Ji W., Yu Y., Li Z., Niu X., Xia W., Lu S. (2018). FGFR1-ERK1/2-SOX2 Axis Promotes Cell Proliferation, Epithelial-Mesenchymal Transition, and Metastasis in FGFR1-Amplified Lung Cancer. Oncogene.

[64] Ji W., Yu Y., Li Z., Wang G., Li F., Xia W., Lu S. (2016). FGFR1 Promotes the Stem Cell-like Phenotype of FGFR1-Amplified Non-Small Cell Lung Cancer Cells through the Hedgehog Pathway. Oncotarget.

[65] Kryczka J., Przygodzka P., Bogusz H., Boncela J. (2017). HMEC-1 Adopt the Mixed Amoeboid-Mesenchymal Migration Type during EndMT. Eur. J. Cell Biol..

[66] Yamada K.M., Sixt M. (2019). Mechanisms of 3D Cell Migration. Nat. Rev. Mol. Cell Biol..

[67] Kryczka J., Papiewska-Pajak I., Kowalska M.A., Boncela J. (2019). Cathepsin B Is Upregulated and Mediates ECM Degradation in Colon Adenocarcinoma HT29 Cells Overexpressing Snail. Cells.

[68] Noordzij I.C., Curvers W.L., Schoon E.J. (2019). Endoscopic Resection for Early Esophageal Carcinoma. J. Thorac. Dis..

[69] Siddik Z.H. (2003). Cisplatin: Mode of Cytotoxic Action and Molecular Basis of Resistance. Oncogene.

[70] Uramoto H., Tanaka F. (2014). Recurrence after Surgery in Patients with NSCLC. Transl. Lung. Cancer Res..

[71] Han M.-L., Zhao Y.-F., Tan C.-H., Xiong Y.-J., Wang W.-J., Wu F., Fei Y., Wang L., Liang Z.-Q. (2016). Cathepsin L Upregulation-Induced EMT Phenotype Is Associated with the Acquisition of Cisplatin or Paclitaxel Resistance in A549 Cells. Acta Pharmacol. Sin..

[72] Shen M., Xu Z., Xu W., Jiang K., Zhang F., Ding Q., Xu Z., Chen Y. (2019). Inhibition of ATM Reverses EMT and Decreases Metastatic Potential of Cisplatin-Resistant Lung Cancer Cells through JAK/STAT3/PD-L1 Pathway. J. Exp. Clin. Cancer Res..

[73] Sarin N., Engel F., Rothweiler F., Cinatl J., Michaelis M., Frötschl R., Fröhlich H., Kalayda G.V. (2018). Key Players of Cisplatin Resistance: Towards a Systems Pharmacology Approach. Int. J. Mol. Sci..

[74] Lima de Oliveira J., Moré Milan T., Longo Bighetti-Trevisan R., Fernandes R.R., Machado Leopoldino A., Oliveira de Almeida L. (2022). Epithelial–Mesenchymal Transition and Cancer Stem Cells: A Route to Acquired Cisplatin Resistance through Epigenetics in HNSCC. Oral Dis..

[75] Yin J., Zhang J. (2011). Multidrug Resistance-Associated Protein 1 (MRP1/ABCC1) Polymorphism: From Discovery to Clinical Application. Zhong Nan Da Xue Xue Bao Yi Xue Ban.

[76] Ween M.P., Armstrong M.A., Oehler M.K., Ricciardelli C. (2015). The Role of ABC Transporters in Ovarian Cancer Progression and Chemoresistance. Crit. Rev. Oncol. Hematol..

[77] Khan P., Bhattacharya A., Sengupta D., Banerjee S., Adhikary A., Das T. (2019). Aspirin Enhances Cisplatin Sensitivity of Resistant Non-Small Cell Lung Carcinoma Stem-like Cells by Targeting MTOR-Akt Axis to Repress Migration. Sci. Rep..

[78] Peng S., Wang J., Lu C., Xu Z., Chai J.-J., Ke Q., Deng X.-Z. (2021). Emodin Enhances Cisplatin Sensitivity in Non-Small Cell Lung Cancer through Pgp Downregulation. Oncol. Lett..

[79] Morelli A.P., Tortelli Jr T.C., Pavan I.C.B., Silva F.R., Granato D.C., Peruca G.F., Pauletti B.A., Domingues R.R., Bezerra R.M.N., De Moura L.P. (2021). Metformin Impairs Cisplatin Resistance Effects in A549 Lung Cancer Cells through MTOR Signaling and Other Metabolic Pathways. Int. J. Oncol..

[80] Duan L., Shen H., Zhao G., Yang R., Cai X., Zhang L., Jin C., Huang Y. (2014). Inhibitory Effect of Disulfiram/Copper Complex on Non-Small Cell Lung Cancer Cells. Biochem. Biophys. Res. Commun..

[81] Lu C., Li X., Ren Y., Zhang X. (2021). Disulfiram: A Novel Repurposed Drug for Cancer Therapy. Cancer Chemother. Pharmacol..

[82] Ntzifa A., Strati A., Kallergi G., Kotsakis A., Georgoulias V., Lianidou E. (2021). Gene Expression in Circulating Tumor Cells Reveals a Dynamic Role of EMT and PD-L1 during Osimertinib Treatment in NSCLC Patients. Sci. Rep..

[83] Rudzinska-Radecka M., Janczewski Ł., Gajda A., Godlewska M., Chmielewska-Krzesinska M., Wasowicz K., Podlasz P. (2021). The Anti-Tumoral Potential of Phosphonate Analog of Sulforaphane in Zebrafish Xenograft Model. Cells.

[84] Wright C., Moore R.D. (1990). Disulfiram Treatment of Alcoholism. Am. J. Med..

[85] Horwitz R., Webb D. (2003). Cell Migration. Curr. Biol..

[86] Zanotelli M.R., Goldblatt Z.E., Miller J.P., Bordeleau F., Li J., VanderBurgh J.A., Lampi M.C., King M.R., Reinhart-King C.A. (2018). Regulation of ATP Utilization during Metastatic Cell Migration by Collagen Architecture. MBoC.

[87] Kang J.H., Lee S.-H., Hong D., Lee J.-S., Ahn H.-S., Ahn J.-H., Seong T.W., Lee C.-H., Jang H., Hong K.M. (2016). Aldehyde Dehydrogenase Is Used by Cancer Cells for Energy Metabolism. Exp. Mol. Med..

[88] Lee J.-S., Lee H., Jang H., Woo S.M., Park J.B., Lee S.-H., Kang J.H., Kim H.Y., Song J., Kim S.-Y. (2020). Targeting Oxidative Phosphorylation Reverses Drug Resistance in Cancer Cells by Blocking Autophagy Recycling. Cells.

[89] Muzio G., Maggiora M., Paiuzzi E., Oraldi M., Canuto R.A. (2012). Aldehyde Dehydrogenases and Cell Proliferation. Free Radic. Biol. Med..

[90] Parajuli B., Fishel M.L., Hurley T.D. (2014). Selective ALDH3A1 Inhibition by Benzimidazole Analogues Increase Mafosfamide Sensitivity in Cancer Cells. J. Med. Chem..

[91] Cocetta V., Ragazzi E., Montopoli M. (2019). Mitochondrial Involvement in Cisplatin Resistance. Int. J. Mol. Sci..

[92] Vesel M., Rapp J., Feller D., Kiss E., Jaromi L., Meggyes M., Miskei G., Duga B., Smuk G., Laszlo T. (2017). ABCB1 and ABCG2 Drug Transporters Are Differentially Expressed in Non-Small Cell Lung Cancers (NSCLC) and Expression Is Modified by Cisplatin Treatment via Altered Wnt Signaling. Respir. Res..

[93] Jolly M.K., Somarelli J.A., Sheth M., Biddle A., Tripathi S.C., Armstrong A.J., Hanash S.M., Bapat S.A., Rangarajan A., Levine H. (2019). Hybrid Epithelial/Mesenchymal Phenotypes Promote Metastasis and Therapy Resistance across Carcinomas. Pharmacol. Ther..

[94] Barr M.P., Gray S.G., Hoffmann A.C., Hilger R.A., Thomale J., O’Flaherty J.D., Fennell D.A., Richard D., O’Leary J.J., O’Byrne K.J. (2013). Generation and Characterisation of Cisplatin-Resistant Non-Small Cell Lung Cancer Cell Lines Displaying a Stem-like Signature. PLoS ONE.

[95] JASP—A Fresh Way to Do Statistics. https://jasp-stats.org/.

[96] Janczewski Ł., Gajda A., Gajda T. (2019). Direct, Microwave-Assisted Synthesis of Isothiocyanates. Eur. J. Org. Chem..

[97] Janczewski Ł., Kręgiel D., Kolesińska B. (2021). Synthesis of Isothiocyanates Using DMT/NMM/TsO− as a New Desulfurization Reagent. Molecules.

[98] Janczewski Ł., Gajda A., Frankowski S., Goszczyński T.M., Gajda T. (2018). T3P^®^—A Benign Desulfurating Reagent in the Synthesis of Isothiocyanates. Synthesis.

[99] Bank R.P.D. RCSB PDB: Homepage. https://www.rcsb.org/.

[100] Fillon M. (2012). Cancer and Natural Selection. J. Natl. Cancer Inst..

