# Supplementary material for: Isothiocyanates (ITCs) 1-(Isothiocyanatomethyl)-4-phenylbenzene and 1-Isothiocyanato-3,5-bis(trifluoromethyl)benzene—Aldehyde Dehydrogenase (ALDH) Inhibitors, Decreases Cisplatin Tolerance and Migratory Ability of NSCLC
Source: Int J Mol Sci. 2022 Aug 3;23(15):8644. doi: 10.3390/ijms23158644 (PMC9369118; doi:10.3390/ijms23158644)
Supplement: Supplementary file 1 [file ijms-23-08644-s001.zip › ijms-1854526-supplementary.pdf]

Supplementary files:

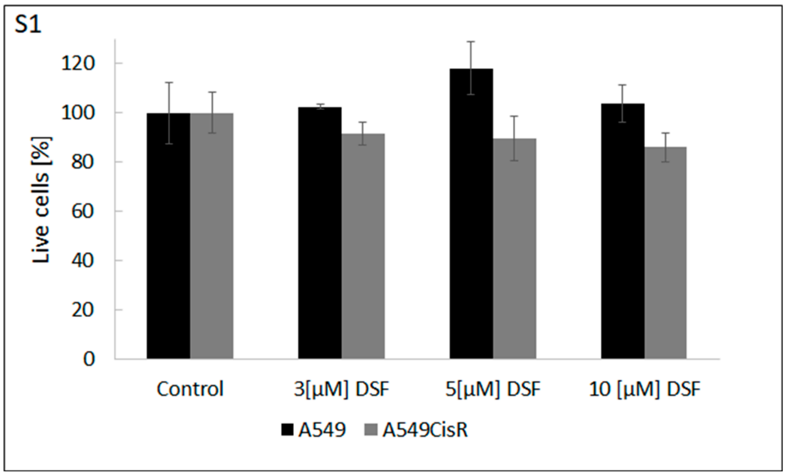

Supplementary S1 *the impact of DSF on A549 and A549CisR cell viability – WST-1 assay after 24h supplementation with different concentration of* ***DSF*** *(ScienCell, Research Lab., Carlsbad, CA, USA).*

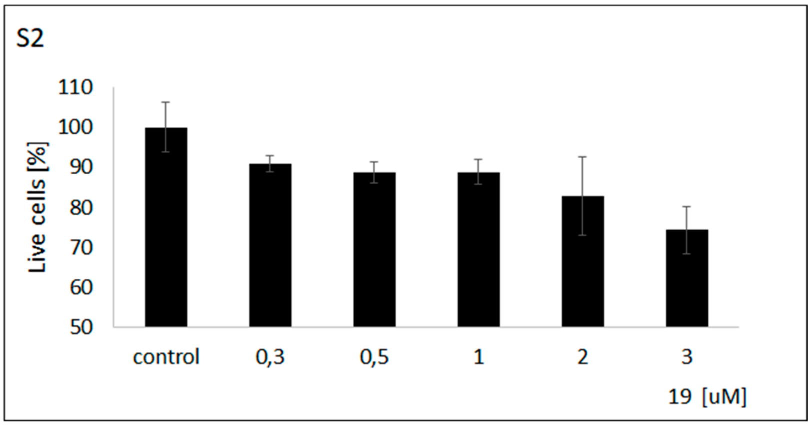

Supplementary S2 *the impact of ITCs 19 on H1581 cell viability – WST-1 assay after 24h supplementation with different concentration of compound* ***19*** *(ScienCell, Research Lab., Carlsbad, CA, USA).*
